# Dysregulation of transition metal ion homeostasis is the molecular basis for cadmium toxicity in *Streptococcus pneumoniae*

**DOI:** 10.1038/ncomms7418

**Published:** 2015-03-03

**Authors:** Stephanie L. Begg, Bart A. Eijkelkamp, Zhenyao Luo, Rafael M. Couñago, Jacqueline R. Morey, Megan J. Maher, Cheryl-lynn Y. Ong, Alastair G. McEwan, Bostjan Kobe, Megan L. O’Mara, James C. Paton, Christopher A. McDevitt

**Affiliations:** 1 Research Centre for Infectious Diseases, School of Biological Sciences, The University of Adelaide, Adelaide, South Australia 4072, Australia; 2 School of Chemistry and Molecular Biosciences, University of Queensland, Brisbane, Queensland 4072, Australia; 3 Australian Infectious Diseases Research Centre, University of Queensland, Brisbane, Queensland 4072, Australia; 4 Institute for Molecular Bioscience, University of Queensland, Brisbane, Queensland 4072, Australia; 5 La Trobe Institute for Molecular Science, La Trobe University, Melbourne, Victoria 3086, Australia; 6 School of Mathematics and Physics, University of Queensland, Brisbane, Queensland 4072, Australia

## Abstract

Cadmium is a transition metal ion that is highly toxic in biological systems. Although relatively rare in the Earth’s crust, anthropogenic release of cadmium since industrialization has increased biogeochemical cycling and the abundance of the ion in the biosphere. Despite this, the molecular basis of its toxicity remains unclear. Here we combine metal-accumulation assays, high-resolution structural data and biochemical analyses to show that cadmium toxicity, in *Streptococcus pneumoniae*, occurs via perturbation of first row transition metal ion homeostasis. We show that cadmium uptake reduces the millimolar cellular accumulation of manganese and zinc, and thereby increases sensitivity to oxidative stress. Despite this, high cellular concentrations of cadmium (~17 mM) are tolerated, with negligible impact on growth or sensitivity to oxidative stress, when manganese and glutathione are abundant. Collectively, this work provides insight into the molecular basis of cadmium toxicity in prokaryotes, and the connection between cadmium accumulation and oxidative stress.

## Abstract (web summary)

The molecular basis for the high toxicity of cadmium is unclear. Here, Begg *et al*. use the bacterium *Streptococcus pneumoniae* as a model system, and show that cadmium uptake increases sensitivity to oxidative stress by reducing intracellular concentrations of manganese and zinc through different mechanisms.

## Graphical abstract

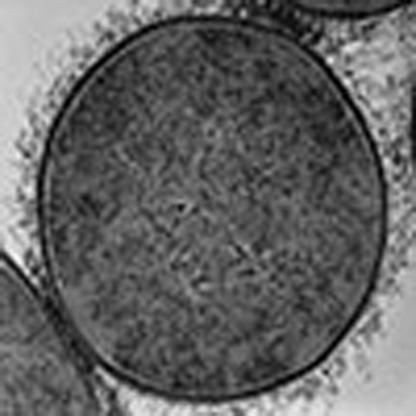

Global cadmium (Cd) production has risen by >1,000-fold since the beginning of the twentieth century to ~20,000 tons per year^1^. Correspondingly, anthropogenic release of Cd into the atmosphere now significantly outstrips natural fluxes and predominantly occurs via non-ferrous ore processing, combustion of fossil fuels, and manufacturing, use and disposal of Cd-containing products^1^. As Cd cannot be degraded, its flux into marine and terrestrial ecosystems has increased the risk of exposure as it accumulates in the food chain, and it is estimated that humans ingest 30 μg of Cd every day^1^,^2^. Cd, which occurs as the divalent cation in the natural environment, is acutely toxic to all forms of life, although there are exceptions to this rule for organisms that have evolved in environments with extremely low zinc abundance^3^. Cd toxicity in humans and microbes is, at a cellular level, closely associated with oxidative stress, despite the inability of Cd^2+^ to directly produce reactive oxygen species^4^. As a consequence, understanding the molecular basis for how Cd^2+^ causes toxicity is a crucial issue.

Cd^2+^ accumulation in microbes and humans primarily occurs by uptake via divalent metal transporters, such as manganese (Mn^2+^) transporters, although the molecular basis for this process remains unknown^5^,^6^. We investigated Cd^2+^ toxicity in the Gram-positive bacterium *Streptococcus pneumoniae* (pneumococcus) as it has only a single Mn^2+^-specific uptake pathway. The pneumococcus acquires Mn^2+^ via the PsaBCA permease, which comprises the ATP-binding cassette (ABC) transporter, PsaBC, and a cell-surface solute-binding protein (SBP), PsaA. The Psa permease is essential for both Mn^2+^ uptake and *in vivo* virulence^7^,^8^,^9^. Similar to other ABC permeases, the SBP PsaA defines the specificity of the uptake pathway. However, our recent studies showed that the functional specificity of the Psa permease in Mn^2+^ acquisition arose not from the specificity of Mn^2+^ binding by PsaA, but instead resulted from the concerted action of protein metal ion coordination chemistry and structural rearrangements that prevented release of other divalent transition metal ions, such as Zn^2+^, into the permease, despite their ability to bind to PsaA^8^. Thus, although it is conceivable that Cd^2+^ may interact with the Psa permease, whether it causes injury to the cell by non-productively competing for metal ion uptake or by accumulation in the cytosol remains unclear.

Here we use a combination of *in vitro*, *in vivo* and structural analyses to investigate how Cd^2+^ impacts transition metal homeostasis in *S. pneumoniae*. We show that *S. pneumoniae* accumulates millimolar concentrations of transition metal ions, and that Cd^2+^ dysregulates metal ion homeostasis by perturbing the Mn^2+^ and Zn^2+^ uptake and efflux pathways, although via strikingly different mechanisms. Manganese accumulation is most affected, and this arises from Cd^2+^ competing with Mn^2+^ ions for uptake via the Psa permease and the concomitant upregulation of the Mn^2+^-efflux pathway. High-resolution structural analyses reveal that although the Mn^2+^-specific Psa permease uses coordination chemistry and structural features to prevent the mistranslocation of other ions, such as Zn^2+^, the larger ionic radius of Cd^2+^ enables this metal to circumvent these regulatory mechanisms. The increased sensitivity to oxidative stress associated with Cd^2+^ toxicity is due to impaired Mn^2+^ acquisition and not directly due to the intracellular accumulation of Cd^2+^ ions, which are buffered on cellular glutathione. Collectively, these findings highlight the challenge that biological systems encounter when non-physiological elements enter the biosphere. This study provides novel insights into the intracellular concentrations of transition metal ions in a Gram-positive cell, the mechanisms associated with their homeostasis, and how Cd^2+^ dysregulates these processes.

## Results

### *S. pneumoniae* accumulates high concentrations of metal ions

**Figure 1:**
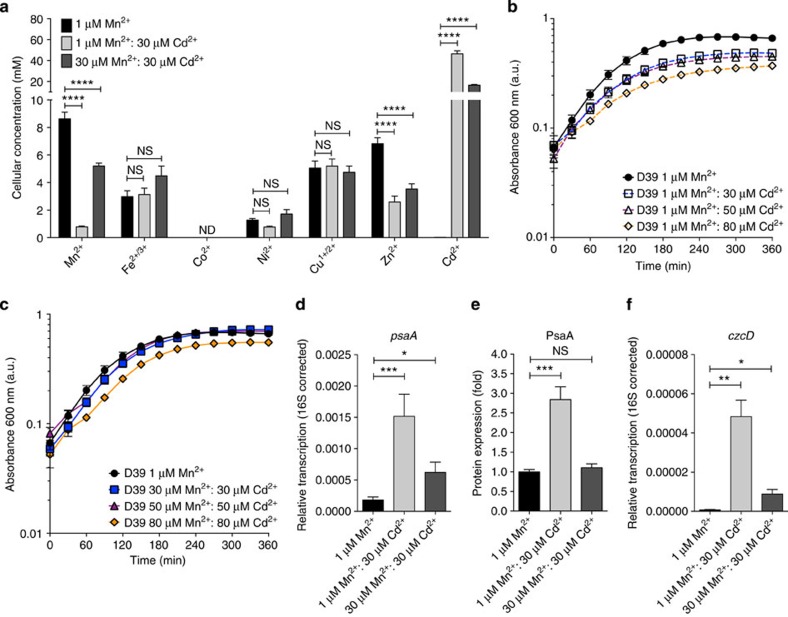
Impact of Cd^2+^ on *S. pneumoniae* growth and first row transition metal homeostasis. (**a**) Total cellular metal ion accumulation of *S. pneumoniae* represented as the mean (±s.e.m.) concentration of ions per cell (determined as c.f.u.) of known cell volume (determined by scanning electron microscopy (SEM)) grown in the specified conditions. The values represent the total cellular metal ion quota, that is, bound, buffered and unbound metal ions, and are from at least three independent biological experiments. Metal ions assessed were Mn^2+^, Fe^2+/3+^, Co^2+^, Ni^2+^, Cu^1+/2+^, Zn^2+^ and Cd^2+^. The statistical significance of the differences in the mean metal concentrations was determined by a one-way ANOVA (ND, not detected; NS, not significant and ****P<0.0001). (**b**,**c**) *S. pneumoniae* (D39) grown in CDM supplemented with metal ions as indicated. The data correspond to mean (±s.e.m.) absorbance at 600 nm measurements from three independent biological experiments. Errors bars, where not visible, are overlapped by the representative symbols. (**d**) Relative transcription, corrected to 16S rRNA, of *psaA* by *S. pneumoniae* when grown in CDM supplemented with 1 μM Mn^2+^ (black), 1 μM Mn^2+^: 30 μM Cd^2+^ (light grey) and 30 μM Mn^2+^: 30 μM Cd^2+^ (dark grey). (**e**) Quantitative immunoblot data of PsaA (Supplementary Fig. 2), from equivalent total protein loadings of whole-cell extracts, from *S. pneumoniae* when grown in CDM supplemented with 1 μM Mn^2+^ (black), 1 μM Mn^2+^: 30 μM Cd^2+^ (light grey) and 30 μM Mn^2+^: 30 μM Cd^2+^ (dark grey). (**f**) Relative transcription, corrected to 16S rRNA, of *czcD* by *S. pneumoniae* when grown in CDM supplemented with 1 μM Mn^2+^ (black), 1 μM Mn^2+^: 30 μM Cd^2+^ (light grey) and 30 μM Mn^2+^: 30 μM Cd^2+^ (dark grey). The data correspond to the mean (±s.e.m.) of three independent biological experiments. The statistical significance of the differences in the mean data was determined by two-tailed unpaired *t*-tests (*P<0.05, **P<0.01 and ***P<0.001).

We first sought to determine the cellular quotient of first row transition metal ions in *S. pneumoniae*. Inductively coupled plasma–mass spectrometry (ICP–MS) data were combined with scanning electron microscopy analyses and colony-forming unit (c.f.u.) counts to derive the intracellular metal ion accumulation data in molar units. In this context, the intracellular metal ion concentration corresponds to the total accumulation of transition metal ions in the cytoplasmic compartment of *S. pneumoniae* and represents all cytoplasmic metal ions, from those bound in tight metal–protein complexes to those occurring as labile pools of exchangeable ions. Although the total number of metal ions per cell was similar to that previously reported for *Escherichia coli*^10^, the pneumococcus has a smaller mean (±s.d.) cell volume, 0.113±0.04 fl (*n*=44; Table 1), and only a single cellular compartment, which results in the total cellular metal ion concentrations being in the millimolar range (Fig. 1a). The Irving–Williams stability series is the near-universal order of metal–protein-binding affinity, which arises from the imperfect steric selection of metal ions that occurs within protein–metal-binding sites. In order of increasing atomic number this can be represented as Mn^2+^<Fe^2+^<Co^2+^<Ni^2+^<Cu^2+^>Zn^2+^ (ref. 11). Our data also revealed that the total cellular quotient of Mn^2+^ (8.6±0.5 mM) was higher than the accumulation of other more competitive ions, such as Zn^2+^ (6.8±0.4 mM). Hence, in *S. pneumoniae*, the accumulation of Mn^2+^ to higher intracellular concentrations may be one factor that aids in increasing its bioavailability, relative to more competitive ions, such as Zn^2+^, and thereby aid in its acquisition by proteins that require Mn^2+^. We then proceeded to ascertain the impact of Cd^2+^ on the cellular accumulation of transition metal ions.

### Cd dysregulates transition metal homeostasis

Titrations of Cd^2+^ into a cation-defined medium (CDM), with a constant concentration of Mn^2+^ (1 μM), showed that pneumococcal growth was perturbed (Fig. 1b and Supplementary Fig. 1a), but that this growth defect could essentially be abrogated by the addition of a stoichiometric concentration of Mn^2+^ to the Cd^2+^-supplemented CDM (Fig. 1c and Supplementary Fig. 1b). *S. pneumoniae* growth in the presence of 30 μM Cd^2+^ reduced the cellular concentrations of Mn^2+^ and Zn^2+^. Manganese accumulation was most affected, with a reduction of ~90% (Fig. 1a; 1 μM Mn^2+^: 30 μM Cd^2+^, *P*<0.0001 (one-way analysis of variance (ANOVA))), despite no significant effect on cell volume (Table 1; 1 μM Mn^2+^: 30 μM Cd^2+^, *P*=0.376 (one-way ANOVA)). Supplementation with Mn^2+^ restored pneumococcal Mn^2+^ accumulation to ~60% of the unchallenged levels (Fig. 1a and Supplementary Table 1; 30 μM Mn^2+^: 30 μM Cd^2+^, *P*<0.0001 (one-way ANOVA)). Zinc also showed a significant decrease in accumulation in the presence of Cd^2+^ (Fig. 1a; 1 μM Mn^2+^: 30 μM Cd^2+^, *P*<0.0001; 30 μM Mn^2+^: 30 μM Cd^2+^, *P*<0.0001 (one-way ANOVA)). Cd^2+^ accumulation increased in the presence of 30 μM Cd^2+^, with or without Mn^2+^ (Fig. 1a). The degree to which Cd^2+^ was accumulated was more than that of any other transition metal ion in the pneumococcus, despite having no physiological role. At 1 μM Mn^2+^: 30 μM Cd^2+^ in the growth medium, intracellular Cd^2+^ concentration was 46.4±2.9 mM. Cd^2+^ accumulation was reduced by nearly 70% on the addition of 30 μM Mn^2+^ to the growth medium (16.5±0.7 mM Cd^2+^), but cellular accumulation of the ion still remained more than that of the other first row transition metal ions.

**Figure 2:**
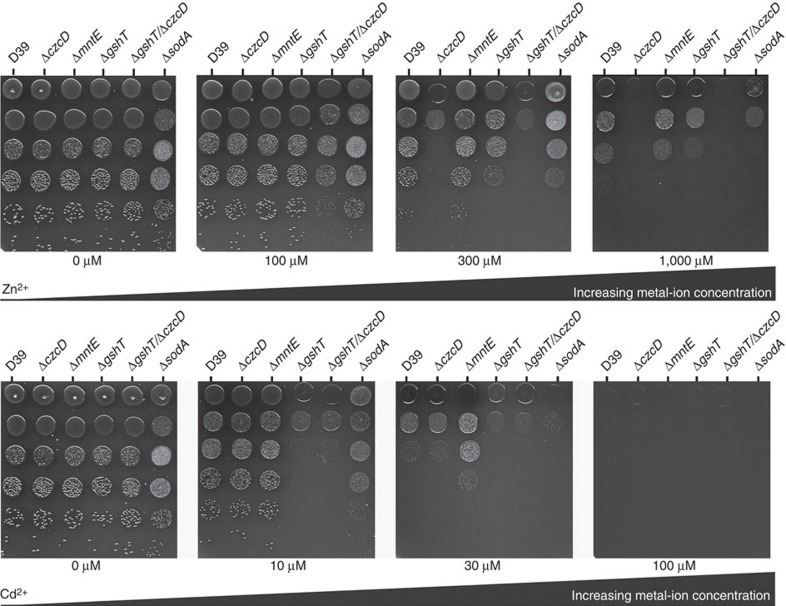
Phenotypic impact of metal ions on *S. pneumoniae* growth. Drop test analysis of *S. pneumoniae* wild-type (D39), Δ*czcD*, Δ*mntE*, Δ*gshT*, Δ*gshT/*Δ*czcD* and Δ*sodA* on blood agar (BA) supplemented with: 0, 100, 300 and 1,000 μM ZnSO_4_ or 0, 10, 30 and 100 μM CdCl_2_. Cells were grown and adjusted to *A*_600_ of 0.4 and were serially diluted. Drops (5 μl) were spotted onto the BA plate, starting from the 10^0^ dilution (top) down to the 10^−5^ dilution (bottom).

We then analysed the impact of Cd^2+^ on transition metal ion homeostatic pathways. Transcriptional analyses revealed that *psaA* transcription increased by 8.4-fold (*P*=0.0007 (two-tailed unpaired *t*-test)) and PsaA expression by 2.8±0.3-fold (*P*<0.0001 (two-tailed unpaired *t*-test)) when grown in the presence of 30 μM Cd^2+^ (Fig. 1d,e), consistent with the Mn^2+^-responsive transcriptional regulator, PsaR, sensing the Mn^2+^ depletion^7^,^12^. Transcription of *psaA* remained elevated during growth supplemented with 30 μM Mn^2+^ albeit to a lesser, but still significant, extent (3.5-fold, *P*=0.0135 (two-tailed unpaired *t*-test)), although PsaA expression did not appear to be significantly increased (Fig. 1d,e). Zinc accumulation in *S. pneumoniae* also occurs via an ABC permease, the Adc permease^9^,^13^,^14^. Transcriptional analysis of the Zn^2+^-responsive regulator, *adcR*^15^, and the Zn^2+^-recruiting SBPs associated with the Adc permease, *adcA* and *adcAII* (refs 13, 14, 16), did not show significant changes in the presence of Cd^2+^ (Supplementary Fig. 3). Despite this, the Zn^2+^ accumulation levels (2.6±0.4 mM) were comparable to strains without a functional Adc permease, Δ*adcCBA* (2.7±0.3 mM) and Δ*adcA/*Δ*adcAII* (2.5±0.3 mM). The perturbation of Zn^2+^ acquisition was not due to competition for the Adc permease as the dominant Zn^2+^-recruiting SBP, AdcA, showed very poor interaction with Cd^2+^ by comparison with Zn^2+^ (Supplementary Fig. 3). Taken together, these findings implicate Cd^2+^ as being able to exert an effect on the Zn^2+^-responsive transcriptional regulators. *S. pneumoniae* uses two Zn^2+^-responsive transcriptional regulators: AdcR, which regulates the Zn^2+^-uptake machinery described above, and SczA, which regulates transcription of the cation diffusion facilitator efflux protein CzcD^15^,^17^. Consequently, we examined whether CzcD was also affected by Cd^2+^ abundance. Growth of *S. pneumoniae* in CDM with 1 μM Mn^2+^: 30 μM Cd^2+^ increased *czcD* transcription by ~70-fold (*P*=0.0011 (two-tailed unpaired *t*-test); Fig. 1f), while supplementation with Mn^2+^ (30 μM Mn^2+^: 30 μM Cd^2+^) reduced this to only ~12.7-fold more than cells grown in unsupplemented CDM (Fig. 1f). Although CzcD from *Cupriavidus metallidurans* is protective against Co^2+^, Zn^2+^ and Cd^2+^ ions^18^, there is no evidence to suggest that the *S. pneumoniae* orthologue has similar poly-specificity. Consequently, we examined an isogenic Δ*czcD* mutant strain to ascertain whether pneumococcal *czcD* contributed to Cd^2+^ resistance. The *S. pneumoniae* Δ*czcD* strain was hypersensitive to extracellular Zn^2+^, consistent with a role in Zn^2+^ homeostasis (Fig. 2). Furthermore, the Cd^2+^-induced efflux of Zn^2+^ observed in wild-type *S. pneumoniae* was abolished in the Δ*czcD* strain (Δ*czcD* 1 μM Mn^2+^, 4.4±0.3 mM Zn^2+^; Δ*czcD* 1 μM Mn^2+^: 30 μM Cd^2+^ 4.2±0.3 mM Zn^2+^; *P*=0.78 (two-tailed unpaired *t*-test)). However, although Cd^2+^ accumulation in the Δ*czcD* strain showed a mild elevation of ~1.4-fold compared with the wild-type, the growth phenotypes of the Δ*czcD* strain were indistinguishable from the wild-type strain and also showed no increase in susceptibility to extracellular Cd^2+^ (Fig. 2 and Supplementary Fig. 4). Taken together, these data indicate that, despite the upregulation of *S. pneumoniae czcD* in response to Cd^2+^ exposure, CzcD is primarily a Zn^2+^ efflux transporter and appears to serve, at best, only a minor protective role against Cd^2+^.

In addition to *czcD*, the pneumococcal genome encodes four other putative metal ion efflux pathways. These are as follows: spd1384 (*mntE*), a paralogue of CzcD involved in Mn^2+^ efflux^19^; spd1438 (*cadD*), a membrane protein of unknown function associated with Cd^2+^ tolerance^20^; two P-type ATPases, spd0635 (*copA*), which is responsible for Cu^1+^ efflux^21^; and spd1927, which has homology to *pmtA*, a PerR-regulated gene in *S. pyogenes* associated with Zn^2+^ resistance^22^. The *mntE* gene has previously been reported to be constitutively expressed and not responsive to cellular Mn^2+^ abundance^19^. Here, we observed that extracellular Cd^2+^ induced a 2.1-fold increase in *mntE* transcription (*P*=0.0211 (two-tailed unpaired *t*-test); Supplementary Fig. 5a). On supplementation with 30 μM Mn^2+^, *mntE* transcription was reduced to non-challenged levels (*P*=0.3665 (two-tailed unpaired *t*-test) Supplementary Fig. 5a), suggesting that intracellular Cd^2+^ concentrations modulated *mntE* expression via an unknown metal-responsive regulator. We then analysed an isogenic Δ*mntE* strain, which lacks the primary Mn^2+^-efflux pathway^19^, for sensitivity to Cd^2+^. Consistent with Cd^2+^ also perturbing Mn^2+^ homeostasis via MntE, the Δ*mntE* strain retained a higher cellular Mn^2+^ concentration (2.53±0.1 mM) than the wild-type (0.78±0.07 mM) (*P*<0.0001 (two-tailed unpaired *t*-test)), when grown in 1 μM Mn^2+^: 30 μM Cd^2+^, and showed greater resistance to extracellular Cd^2+^ (Fig. 2). The *cadD* gene showed a similar trend to *czcD* and *mntE* with a ~3.6-fold increase in transcription when challenged with 30 μM Cd^2+^ (*P*<0.0001 (two-tailed unpaired *t*-test); Supplementary Fig. 5b), followed by abrogation when supplemented with 30 μM Mn^2+^. However, the P-type ATPases, *copA* and spd1927, were transcriptionally unresponsive to Cd^2+^ (Supplementary Fig. 5c,d), suggesting that they were unlikely to contribute to Cd^2+^ management. Collectively, these data show that Cd^2+^ accumulation was associated with transcriptional activation of several metal ion membrane protein transporters. However, of those pathways with known efflux function, that is, CzcD and MntE, Cd^2+^ ions subverted their primary functional roles and, instead, further dysregulated transition metal ion homeostasis in *S. pneumoniae* via amplification of Zn^2+^ and Mn^2+^ efflux, respectively. The mechanism(s) of Cd^2+^ efflux in *S. pneumoniae*, if any, remains to be identified.

### Cd^2+^ competes with Mn^2+^ for the Psa permease

**Figure 3:**
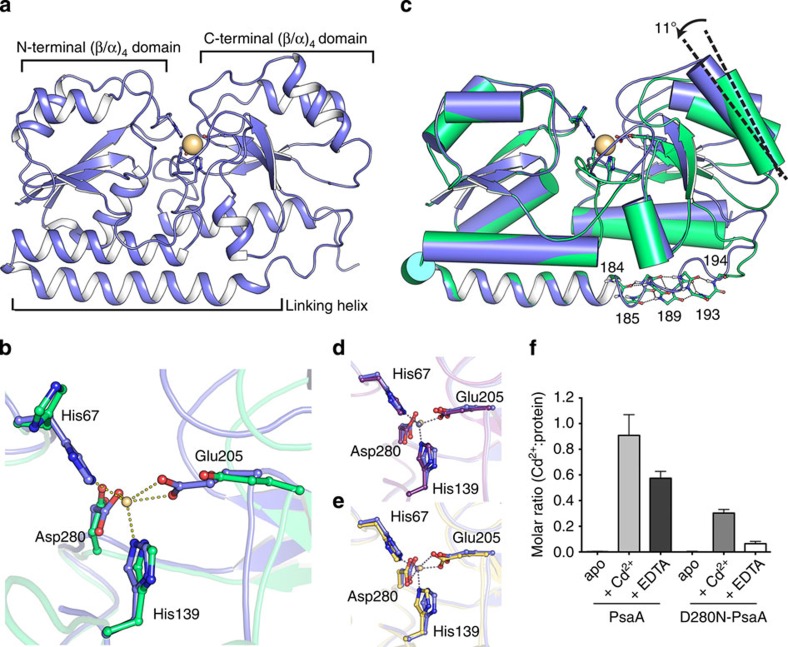
Structure of Cd^2+^-bound PsaA. (**a**) Cartoon illustration of Cd^2+^-bound wild-type PsaA in closed conformation (PDB accession code 4UTP). The structure consists of N-terminal and C-terminal (β/α)_4_ domains with a domain-linking helix. The metal-binding site is situated between the two domains, in which a Cd^2+^ ion (pale-yellow sphere) is bound. (**b**) The metal-binding site of wild-type PsaA in the presence (light blue, PDB accession code 4UTP) and absence (green, PDB accession code 3ZK7) of Cd^2+^ (pale-yellow sphere), with the metal coordination shown as dashed lines. (**c**) Superposition of Cd^2+^-bound (light blue, PDB accession code 4UTP) and apo (green, PDB accession code 3ZK7) wild-type PsaA structures (Cα atoms, residues 32–163, were used for the superposition). The linking helix is shown in cartoon representation, with other helices shown as cylinders. The crossing angle of the C-terminal (β/α)_4_ domains between the structures is marked. The bound Cd^2+^ ion is shown as a pale-yellow sphere. In the apo structure, backbone atoms in the flexible region of the linking helix (residues 184–194) are shown in stick representation, with hydrogen bonds between backbone atoms as dashed lines. (**d**) Comparison of the metal-binding sites between Cd^2+^-bound (light blue, PDB accession code 4UTP) and Zn^2+^-bound (purple, PDB accession code 1PSZ) wild-type PsaA structures. The metal ions are shown as spheres (Cd^2+^, pale yellow; Zn^2+^, grey) and their coordination as dashed lines. (**e**) Comparison of the metal-binding sites between Cd^2+^-bound (light blue, PDB accession code 4UTP) and Mn^2+^-bound (yellow, PDB accession code 3ZTT) wild-type PsaA structures. The metal ions are shown as spheres (Cd^2+^, pale yellow; Mn^2+^, blue) and their coordination as dashed lines. In **a**–**e**, the metal-coordinating residues are shown in stick representation. (**f**) *In vitro* metal binding of Cd^2+^ to wild-type or D280N variant apo-PsaA analysed by ICP–MS. Data correspond to the mean (±s.d.) molar ratio of Cd^2+^ to the PsaA isoform for apo-PsaA (black), Cd^2+^-PsaA (light grey), EDTA-Cd^2+^-PsaA (dark grey). apo-D280N-PsaA (black), Cd^2+^-D280N-PsaA (grey) and EDTA-Cd^2+^-D280N-PsaA (white).

To elucidate the hitherto unexplained molecular basis for Cd^2+^ uptake via Mn^2+^ transporters, we examined the interaction of Cd^2+^ with the Mn^2+^-recruiting SBP PsaA. Although the physiological role of PsaA is in recruiting Mn^2+^, the metal-binding site of the protein has previously been shown to interact with other divalent transition metal ions^8^. However, coordination chemistry at the metal-binding site and structural dynamics prevent release of these other ions, and thereby maintain the functional role of the Psa permease in Mn^2+^ uptake^8^. Here the interaction of Cd^2+^ with PsaA was analysed by determining the structure of wild-type PsaA with Cd^2+^ at 2.0 Å resolution (Fig. 3a and Supplementary Fig. 6). The PsaA–Cd^2+^ complex revealed that Cd^2+^ was coordinated by the metal-binding site residues His67, His139, Glu205 and Asp280 in a structure that was highly similar (root-mean-square deviation (RMSD)<0.3 Å over 242 Cα atoms) to our previously reported Mn^2+^- and Zn^2+^-bound structures^23^,^24^. Nevertheless, small re-orientations of the side chains of the metal-coordinating residues allowed the bulkier Cd^2+^ ion (ionic radius of 95 pm for Cd^2+^ versus 84 pm for Mn^2+^) to be accommodated into the protein–metal-binding site (Fig. 3b and Supplementary Table 2). In the PsaA–Cd^2+^ structure, the distances between metal-coordinating atoms and the metal ion were increased by ~0.2 Å compared with those for PsaA-Zn^2+^ (Supplementary Table 2). Similar increases in metal–protein distances have been observed for the Cd^2+^–thiolate-binding sites of Cd^2+^-substituted metallothionein and rubredoxin proteins, compared with the Zn^2+^-bound equivalents^25^,^26^. Although Cd^2+^ and Zn^2+^ often display similar coordination spheres in proteins, the longer bond distances observed for Cd^2+^ usually result in an increase in the coordination number. Accordingly, the coordination geometry in the PsaA–Cd^2+^ structure is an intermediate between a square-pyramid and trigonal-bipyramid (*n*=5), in contrast to the 4- and 6-coordinate sites observed for the Zn^2+^- and Mn^2+^-bound PsaA structures, respectively (Fig. 3d,e and Supplementary Table 3). A comparison of the observed coordination of PsaA–Cd^2+^ with other metal-binding sites of metalloprotein structures in the PDB, composed of mixed N/O ligating atoms, revealed that Cd^2+^ is found in a variety of coordination geometries with coordination numbers ranging from 5 to 7 (Supplementary Table 4). Nevertheless, the only bona fide protein–metal-binding site for Cd^2+^ is in carbonic anhydrase from *Thalassiosira weissflogii*, which has been shown to be a cambialistic enzyme with Zn^2+^ in a tetrahedral geometry and Cd^2+^ in a trigonal-bipyramidal geometry^3^,^27^,^28^. Thus, the coordination geometry found in PsaA–Cd^2+^ conforms to what has been observed for the only known naturally occurring Cd^2+^-binding protein, and for other protein structures deposited in the PDB. We further examined the interaction of metal-free (apo) PsaA with Cd^2+^ and observed that apo-PsaA bound Cd^2+^ in a 1:1 molar ratio (Fig. 3f), with a *K*_D_ of 5.6±1 nM (Supplementary Fig. 7). As the cognate ligand, Mn^2+^, has a *K*_D_ of 3.3±1 nM for PsaA, the relatively small (approximately twofold) difference in the *K*_D_s is consistent not only with the competitive effect on Mn^2+^ acquisition but also the continued, albeit reduced, Cd^2+^ accumulation during growth in the presence of equimolar concentrations of both ions (30 μM Mn^2+^: 30 μM Cd^2+^). Collectively, these findings are consistent with the observed impact of Cd^2+^ on Mn^2+^ accumulation in the wild-type and mutant *S. pneumoniae* strains and indicate that it is a competitive effect mediated via PsaA. However, they also indicate that interaction of Cd^2+^ with PsaA is distinct from the previously characterized Mn^2+^ and Zn^2+^ interactions.

### PsaA is permissive for Cd^2+^ binding and release

**Figure 4:**
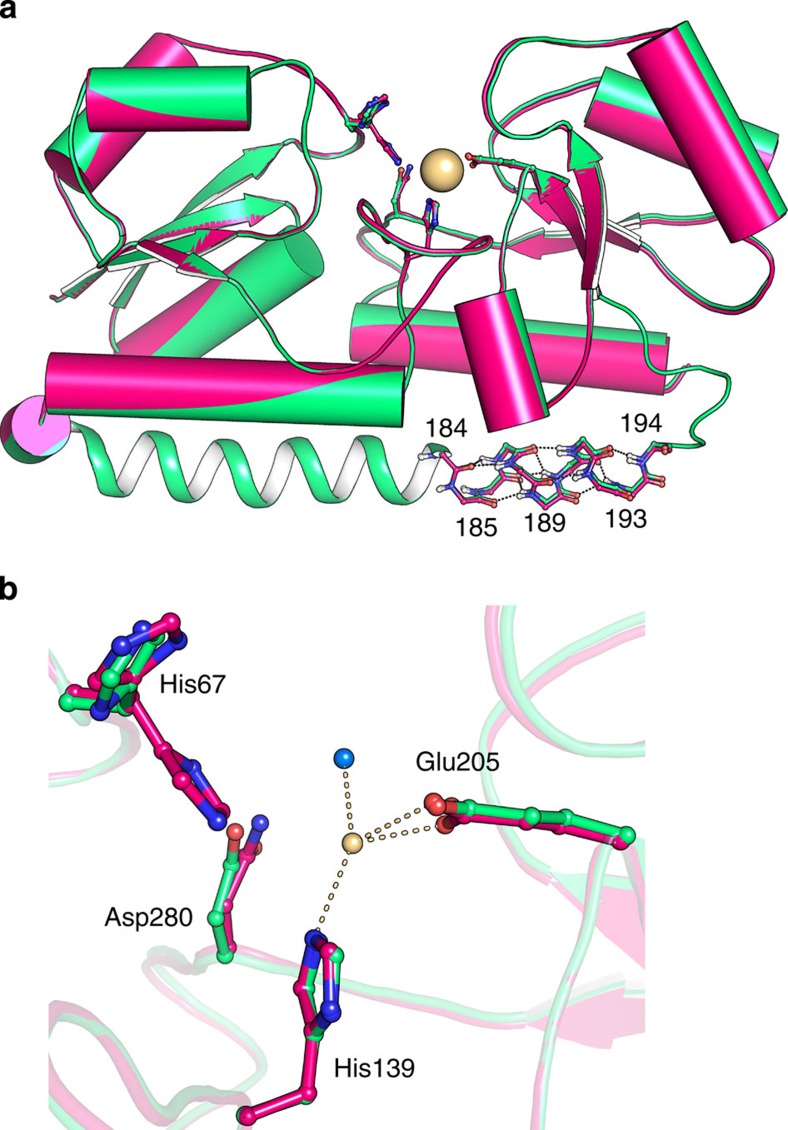
Structure of Cd^2+^-bound PsaA-D280N in an open conformation. (**a**) Superposition of Cd^2+^-bound PsaA-D280N (magenta, PDB accession code 4UTO) and apo wild-type (green, PDB accession code 3ZK7) PsaA structures (Cα atoms, residues 32–163, were used for the superposition). The bound Cd^2+^ ion is shown as a pale-yellow sphere. The linking helix is shown in cartoon representation (up to residue 183) and backbone atoms in the flexible region of the linking helix (residues 184–194) are shown in stick representation, with hydrogen bonds between backbone atoms as dashed lines. (**b**) Close-up view of the metal-binding sites between the superimposed Cd^2+^-bound PsaA-D280N (magenta, PDB accession code 4UTO) and apo wild-type (green, PDB accession code 3ZK7) PsaA structures. The bound Cd^2+^ ion is shown as a pale-yellow sphere and its coordination with the protein residues is shown as dashed lines. The water molecule coordinating the metal is shown as a light-blue sphere. In **a**,**b**, the metal-coordinating residues are shown in stick representation.

PsaA uses a ‘spring-hammer’-like mechanism for metal ion binding, in which the dynamics of the metal ion-loaded protein are dictated by a combination of metal ion geometry and the distortion of a helix linking the N- and C-terminal lobes of the protein^8^. We further analysed the metal-binding site coordination geometry by use of an Asp280 variant isoform (PsaA-D280N), as it would be incapable of facilitating the trigonal-bipyramidal coordination observed in wild-type PsaA^8^. The structure of PsaA-D280N bound to Cd^2+^, refined to 1.7 Å resolution, revealed a partially solvated Cd ion loosely interacting with residue Glu205 (Fig. 4a,b). The C-terminal lobe of PsaA showed minor movement (~1.0° relative to the open, metal-free conformation), but the inability of Cd^2+^ to ligate to residue Asp280 prevented further conformational changes. Consistent with these observations, PsaA-D280N was greatly impaired in Cd^2+^ binding (Fig. 3f).

We then assessed the distortion of the linking helix by monitoring the number of disrupted backbone hydrogen bonds in its flexible region (residues 184–194) in wild-type PsaA–Cd^2+^ (Figs 3c and 4a, and Supplementary Table 5)^8^. Both the hinge-bending angle between the N- and C-terminal lobes and the number of main-chain hydrogen bonds disrupted in the residue range 184–194 corresponded more closely to the Mn^2+^-bound structure than to the ‘locked’ Zn^2+^-structure. This indicated that Cd^2+^ binding was a reversible process (Supplementary Table 5), consistent with the observation that Cd^2+^ could be extracted (~40%) from the PsaA–Cd^2+^ complex by using the chelating agent EDTA (Fig. 3f). Thus, these findings provide further support for the conclusion that both Mn^2+^ and Cd^2+^ compete for transport via the Psa permease, consistent with the cellular accumulation data, which shows that Mn^2+^ uptake occurs at the expense of Cd^2+^ import and vice versa.

### Reduced Mn^2+^ levels result in sensitivity to oxidative stress

**Figure 5:**
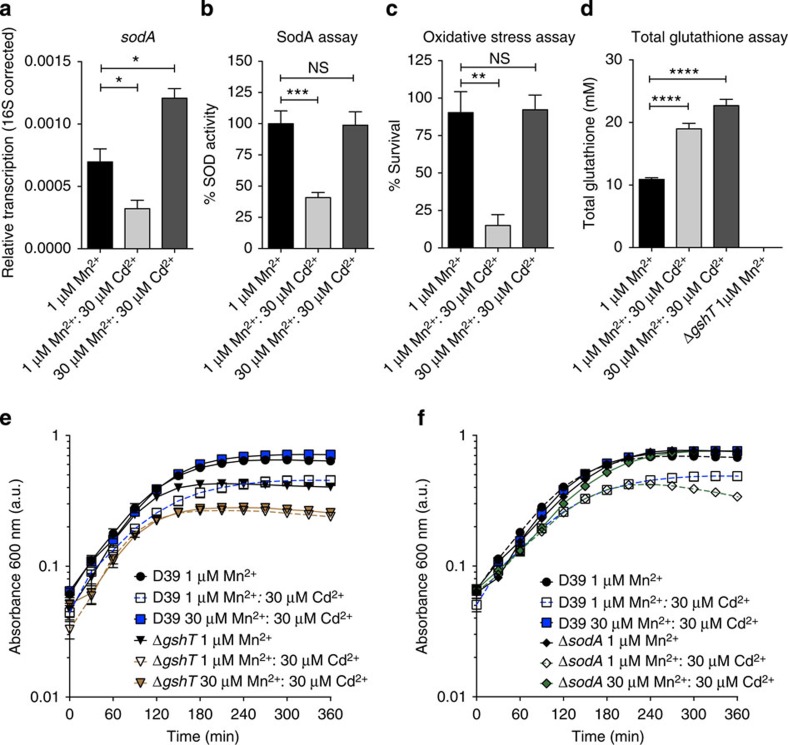
The impact of Cd^2+^ on sensitivity to oxidative stress. (**a**) Relative transcription, corrected to 16S rRNA, of *sodA* by *S. pneumoniae* when grown in CDM supplemented with 1 μM Mn^2+^ (black), 1 μM Mn^2+^: 30 μM Cd^2+^ (light grey) and 30 μM Mn^2+^: 30 μM Cd^2+^ (dark grey). The data correspond to the mean (±s.e.m.) of three independent biological experiments. (**b**) SodA activity assay *S. pneumoniae* when grown in CDM supplemented with 1 μM Mn^2+^ (black), 1 μM Mn^2+^: 30 μM Cd^2+^ (light grey) and 30 μM Mn^2+^: 30 μM Cd^2+^ (dark grey). Activity was calculated as a percentage of SodA activity observed for *S. pneumoniae* cells grown in 1 μM Mn^2+^. The data correspond to the mean (±s.e.m.) of three independent biological experiments. (**c**) Paraquat killing of *S. pneumoniae* grown in CDM supplemented with 1 μM Mn^2+^ (black), 1 μM Mn^2+^: 30 μM Cd^2+^ (light grey) and 30 μM Mn^2+^: 30 μM Cd^2+^ (dark grey). Survival was calculated as a percentage of colonies at 30 min compared with cells not challenged with paraquat. The data correspond to the mean (±s.e.m.) of three independent biological experiments. (**d**) Total glutathione determination per mean cell volume for *S. pneumoniae* grown in CDM supplemented with 1 μM Mn^2+^ (black), 1 μM Mn^2+^: 30 μM Cd^2+^ (light grey) and 30 μM Mn^2+^: 30 μM Cd^2+^ (dark grey). The statistical significance of the differences in the mean data for **a**–**d** was determined by two-tailed unpaired *t*-tests (NS, not significant; *P<0.05, **P<0.01, ***P<0.001, ****P<0.0001). (**e**,**f**) *S. pneumoniae* Δ*gshT* (**e**) and Δ*sodA* (**f**) grown in CDM supplemented with metal ions as indicated. The data correspond to mean (±s.e.m.) absorbance 600 nm measurements from three independent biological experiments. Errors bars, where not visible, are overlapped by the representative symbols.

We then sought to assess how Cd^2+^ affected oxidative stress management in *S. pneumoniae*. Manganese has a prominent role in regulating the expression of superoxide dismutase (*sodA*), where it also serves as a cofactor^29^,^30^,^31^. Transcription of *sodA* was decreased under Cd^2+^-induced Mn^2+^ starvation, but this was restored when supplemented with Mn^2+^ (Fig. 5a), and direct measurement of SodA activity from *S. pneumoniae* showed a similar trend (Fig. 5b). The impact of Cd^2+^ on oxidative stress response was ascertained using paraquat, which causes oxidative damage by promoting a futile redox cycle in the cytoplasm. We observed that although growth in the presence of Cd^2+^ resulted in a significant decrease in survival (Fig. 5c, *P*=0.0087 (two-tailed unpaired *t*-test)), the increased sensitivity was not due to Cd^2+^, but instead was a result of the decreased Mn^2+^ accumulation, as *S. pneumoniae* grown in 30 μM Mn^2+^: 30 μM Cd^2+^ exhibited wild-type levels of survival (Fig. 5c). Hence, the perturbation of Mn^2+^ homeostasis by Cd^2+^ heightens the sensitivity to oxidative stress, while Cd^2+^ could be accumulated to high intracellular concentrations with no apparent deleterious effect. This ability to accumulate Cd^2+^ without direct toxicity indicated that intracellular buffering of the ion was crucial.

Reduced thiol groups on small peptides, primarily glutathione in *S. pneumoniae*, have been implicated in having an essential role in intracellular transition metal ion management (Fe^2+^ and Zn^2+^) and in ameliorating Cd^2+^ toxicity^32^. *S. pneumoniae* is incapable of *de novo* glutathione synthesis and acquires glutathione via a high-affinity ABC permease, the Gsh permease^32^. Here we investigated the contribution of glutathione to management of transition metal ion stress. In *S. pneumoniae*, the total glutathione content, in response to Cd^2+^ exposure, increased by approximately twofold to a mean (±s.e.m.) pneumococcal cell concentration of 19.0±0.9 mM (Fig. 5d). Glutathione abundance is crucial for Cd^2+^ management, as a mutant strain incapable of glutathione acquisition, *S. pneumoniae* Δ*gshT*, was hypersensitive to Cd^2+^ stress (1 μM Mn^2+^: 30 μM Cd^2+^; Figs 2 and 5e) and, in contrast to the wild type, could not be rescued by addition of Mn^2+^ (Fig. 5e; 30 μM Mn^2+^: 30 μM Cd^2+^). By contrast, the Δ*sodA* strain, which we have previously shown to be hypersensitive to oxidative stress challenge^30^,^33^, demonstrated a wild-type growth phenotype in the presence of Cd^2+^, with no apparent increase in sensitivity to metal ion stress (Figs 2 and 5f). Taken together, these findings indicate that glutathione serves a major role in Cd^2+^ buffering, whereas SodA appears to be dispensable, consistent with the lack of redox activity of Cd^2+^ ions.

To ascertain whether this was also the case for Zn^2+^, which is also presumed to be buffered by glutathione, we examined the response of the Δ*gshT* strain to Zn^2+^ exposure. Intriguingly, while the Δ*gshT* strain was insensitive to subinhibitory concentrations of Zn^2+^, similar to the Δ*czcD* strain, the combination mutant (Δ*gshT*/Δ*czcD*) showed significant attenuation of growth (100 μM Zn^2+^; Fig. 2). At higher concentrations of Zn^2+^, the growth of the Δ*gshT*/Δ*czcD* strain corresponded with the hypersensitive phenotype of the Δ*czcD* strain, while the Δ*gshT* strain showed a very mild impact, relative to wild type (300 μM Zn^2+^; Fig. 2). Taken together, these data show that intracellular management of Zn^2+^ occurs via the concerted actions of both glutathione and Zn^2+^ efflux, wherein the activation and efflux via CzcD is crucial to prevent Zn^2+^ intoxication. By contrast, management of Cd^2+^, which has no major efflux pathway, is predominantly dependent on cellular glutathione to prevent mismetallation of other proteins. Concordantly, when Cd^2+^ accumulation surpasses the buffering capacity of the glutathione pool (1 μM Mn^2+^: 30 μM Cd^2+^), or in the absence of intracellular glutathione (Δ*gshT*), this leads to Cd^2+^ toxicity.

## Discussion

Anthropogenic-facilitated entry of non-physiological elements, such as Cd^2+^, into the biosphere presents unique cellular challenges for biological systems. The prior absence of these elements from the biosphere obviated any selective pressure towards evolving mechanisms for managing their impact on biological processes. As a consequence, many non-physiological elements rapidly accumulate in the food chain leading to significant toxicity in higher organisms^1^,^2^. We observed that Cd^2+^ was toxic to *S. pneumoniae* and that this arose from acute dysregulation of the transition metal ion homeostatic mechanisms. Elucidation of the total cellular quotient of metal ions in *S. pneumoniae* not only showed the impact of Cd^2+^ but also revealed novel aspects of cellular transition metal homeostasis. *S. pneumoniae* is a Gram-positive organism, comprising only a single cellular compartment; thus the concentration of transition metal ions accurately reflects their total intracellular accumulation and encompasses metal ions occurring in labile exchangeable pools and those bound weakly or tightly to proteins, peptides, nucleic acids and other molecules. Determination of the cellular quotient of metal ions in wild-type *S. pneumoniae* and mutant variants revealed the range over which cellular variation of transition metal ion concentrations is permissible. Manganese accumulation shows significant plasticity, with cells being viable at concentrations as low as ~3% of typical accumulation levels^23^,^30^. By contrast, Zn^2+^ accumulation is regulated in a much narrower window, with its minimal cellular quotient being between 20 and 40% of typical accumulation. Intriguingly, the higher total cellular quotient of Mn^2+^ ions suggests that the concentration ‘set-points’ for cellular accumulation of transition metal ions may also contribute to ensuring efficient acquisition of metal cofactors by proteins. Manganese, which has been reported to be buffered by molecules such as citrate, phosphate and histidines residues from proteins in the cytoplasm^34^,^35^, has fewer reported cellular roles. Hence, its presence at such high cellular concentrations suggests that in *S. pneumoniae* it could occur predominantly as a labile pool of cytoplasmic-buffered Mn^2+^. By contrast Zn^2+^, which is reported in numerous studies to be highly utilized in intracellular proteins^36^,^37^, has been predicted to have a significantly smaller labile pool^10^,^36^. Comprehensive intracellular metal ion speciation studies have been limited by a combination of technical and methodological challenges that remain to be surmounted. However, the data here allow us to speculate that, in *S. pneumoniae*, the potentially larger labile pool of Mn^2+^ ions, relative to Zn^2+^ ions, could be one mechanism that aids in Mn^2+^-dependent proteins correctly acquiring their cognate metal cofactor. The millimolar cellular quotient of Zn^2+^ ions in *S. pneumoniae* may also provide a plausible basis for the unexplained lower sensitivity of the zinc-responsive transcriptional regulator AdcR to Zn^2+^ ions, when compared with the homoplastic regulator, Zur, from *E. coli*^10^,^15^. In *E. coli*, Zn^2+^ has been shown to accumulate to an overall (cytoplasmic plus periplasmic) cellular concentration of ~0.2 mM and Zur is highly sensitive to Zn^2+^ abundance, sensing Zn^2+^ ions in the femtomolar range^10^. By contrast, AdcR is 2–3 orders of magnitude less sensitive, suggesting that a higher cellular ‘set-point’ for sensing of Zn^2+^ ions in *S. pneumoniae* may exist^15^.

In the context of exposure to Cd^2+^, the impact on transition metal ion homeostasis corresponds to specific dysregulation of Mn^2+^ and Zn^2+^ accumulation, but via different mechanisms. Manganese accumulation was disrupted by direct competition of Cd^2+^ for the Psa permease and the upregulation of the Mn^2+^-efflux pathway MntE. These results also suggest that PsaR, the transcriptional regulator of the Psa permease, is not functionally activated by Cd^2+^ and thus permits upregulation of *psaBCA*. In this way, the interaction of Cd^2+^ with PsaR may mimic the binding of Zn^2+^ rather than Mn^2+^, which occurs in a manner that does not permit repression of *psaBCA* transcription^12^. Although competition for the Psa permease was consistent with expectations, based on the inability of the SBP to be specific for solely Mn^2+^ ions, the mistranslocation of Cd^2+^ ions via the permease highlights an evolutionary limitation in managing extracellular stress by non-physiological elements. Specific acquisition of Mn^2+^ via the Psa permease arises from a combination of mechanisms that prevent the transport of Zn^2+^ ions, which are more abundant in the native niche of *S. pneumoniae*. However, the larger ionic radius and ‘strained’ coordination of the Cd^2+^ ion at the metal-binding site, similar to Mn^2+^ (Supplementary Table 3), resulted in an easily destabilized metal–protein complex, such that PsaA was incapable of preventing release of Cd^2+^ ions into the permease. This underscores the challenge encountered by metalloregulatory mechanisms when interacting with elements that have only recently entered the biosphere. In PsaA, the regulatory mechanisms that have evolved to prevent mistranslocation of Zn^2+^ ions are not capable of preventing the translocation of Cd^2+^ ions. By contrast with Mn^2+^, the Cd^2+^-induced disruption of Zn^2+^ homeostasis occurred via upregulation of *czcD*, the Zn^2+^-efflux pathway, and the apparent inability of the pneumococcus to transcriptionally activate additional Zn^2+^-recruiting proteins (that is, AdcAII (ref. 13)) to compensate for the depletion of cellular Zn^2+^ ions. Hence, Cd^2+^ accumulation activated the coordinated pneumococcal physiological response to Zn^2+^ intoxication or overload^13^,^15^,^17^,^23^, resulting in the futile export and depletion of cellular Zn^2+^. Our findings suggest that Cd^2+^ accumulation results in the mismetallation of the Zn^2+^-responsive transcriptional regulators, AdcR and SczA^13^,^15^,^17^. Whether this is due to Cd^2+^ directly interacting with the Zn^2+^-binding sites of intracellular metal sensors^38^,^39^,^40^ or via Cd^2+^-mediated displacement of Zn^2+^ from thiol buffering sites^41^, which results in the non-physiological activation of the Zn^2+^-responsive transcriptional regulators, remains to be determined. Irrespective of this fact, transcriptional activation of *czcD* is often construed as a mechanism to facilitate Cd^2+^ efflux based on studies of the archetypal protein from *C. metallidurans*^18^. However, in *S. pneumoniae*, similar to *S. pyogenes*^42^, CzcD is primarily a Zn^2+^-efflux pathway and does not provide a major protective role against Cd^2+^ ions. As a consequence, in *S. pneumoniae*, the Cd^2+^-induced upregulation of *czcD* appears to preferentially deplete intracellular Zn^2+^, despite the greater abundance of Cd^2+^ ions.

Cd^2+^ accumulation is associated with oxidative stress, despite the redox-inert nature of the ion^4^. The data here show that, in *S. pneumoniae*, the increased sensitivity to oxidative stress arises from the loss of *sodA* expression and activity due to Cd^2+^-induced Mn^2+^ depletion. However, the loss of SodA was an indirect by-product of Cd^2+^ accumulation and, importantly, not associated with the ability of the pneumococcus to tolerate Cd^2+^ ions. This was demonstrated by the Δ*sodA* strain, which showed wild-type growth phenotypes in the presence of extracellular Cd^2+^, and in wild-type *S. pneumoniae*, which retained the ability to accumulate high cellular concentrations of Cd^2+^ without an increase in sensitivity to oxidative stress, when supplemented with Mn^2+^. However, SodA still has a major role in the *in vitro* and *in vivo* fitness of *S. pneumoniae* owing to a lack of other mechanisms to detoxify superoxide^23^,^30^,^33^. Here, in the presence of Cd^2+^, the loss of SodA also occurs in conjunction with dysregulation of Mn^2+^ and Zn^2+^ homeostatic mechanisms and the accumulation of very high concentrations of Cd^2+^. Hence, it is the combination of these factors that compromises pneumococcal viability in the presence of Cd^2+^.

Intracellular accumulation of Cd^2+^ in *S. pneumoniae* was crucially dependent on glutathione, which acts as a low-affinity metal ion buffer for certain transition metal ions (Fe^2+^, Zn^2+^ and Cd^2+^), as phenotypic growth perturbations were seen when Cd^2+^ accumulation exceeded the total cellular glutathione pool (19–23 mM). Although it cannot be completely discounted that glutathione contributes to pneumococcal oxidative stress management^32^, the hypersensitivity of the Δ*gshT* strain to extracellular Cd^2+^, in contrast to the Δ*sodA* strain, strongly implicates the major role for cellular glutathione is in metal ion buffering. The observed hypersensitivity of the Δ*gshT*/Δ*czcD* strain to Zn^2+^ stress further underscores how intracellular metal ion homeostasis relies on the contributions of both cellular glutathione and the efflux machinery^13^,^43^. The results are also consistent with the central role of low-molecular-weight thiols for metal ion homeostasis in other microorganisms. Bacillithiol has recently been shown to have a crucial role in the buffering of Zn^2+^ ions in *Bacillus subtilis*^41^, while it also serves an undefined, but protective role in Cd^2+^ stress for *B. subtilis* and *Staphylococcus aureus*^44^. Although *S. pneumoniae* and other microorganisms lack a bacillithiol biosynthetic pathway, the role of low-molecular-weight species for metal ion homeostasis is emerging as a crucial mechanism. Overall, our findings here highlight the role of glutathione as an intracellular metal ion buffer, in preference to oxidative stress management. This finding has broad ramifications for the assumed role(s) of cellular glutathione, and related thiol derivatives, in prokaryotes and potentially eukaryotes^43^,^41^.

In conclusion, Cd^2+^ accumulation in the food chain and its toxicity are crucial, but largely overlooked concerns. Our findings reveal the molecular basis by which Cd^2+^ is mistranslocated via a Mn^2+^ transporter and provides new insights into how Cd^2+^ dysregulates intracellular transition metal homeostasis. Disruption of multiple essential metal ion homeostatic mechanisms is highly damaging to biological organisms, such as *S. pneumoniae*^14^, and in this way Cd^2+^ acts to severely dysregulate both Mn^2+^ and Zn^2+^ homeostasis (Supplementary Fig. 8). By revealing the remarkably high (millimolar) cellular quotients of transition metal ions and glutathione, this work also shows how Cd^2+^, despite its inability to directly generate reactive oxygen species, is associated with oxidative stress. This work provides a new understanding of the mechanisms by which Cd^2+^ enters cells and causes toxicity, and thereby, opens the way to identifying new routes towards developing specific therapeutic agents capable of preventing Cd^2+^ toxicity.

## Methods

### Growth experiments and whole-cell assays

The *S. pneumoniae* D39 Δ*sodA*, Δ*czcD*, Δ*gshT*, Δ*mntE*, Δ*adcCBA* and Δ*adcA/*Δ*adcAII* strains have been generated previously^13^,^32^. The Δ*mntE* and Δ*gshT*/Δ*czcD* strains were generated as described previously^13^, using primer sequences listed in Supplementary Table 6. *S. pneumoniae* D39, Δ*sodA*, Δ*czcD*, Δ*mntE*, Δ*gshT*, Δ*gshT*/Δ*czcD*, Δ*adcCBA* and Δ*adcA/*Δ*adcAII* were grown in CDM, which corresponded to the C+Y media without transition metal ion supplementation^23^. The base ion content of the CDM was ascertained by ICP–MS on an Agilent 7500cx ICP–MS (Adelaide Microscopy, University of Adelaide)^23^. Growth experiments were conducted in CDM supplemented with 1 μM MnSO_4_ and concentrations of 10, 30, 50 or 80 μM of CdCl_2_ or MnSO_4_, as specified. All chemicals used in this study were purchased from Sigma-Aldrich, unless otherwise specified. For bacterial growth experiments, an inoculum was prepared from overnight grown culture on a blood-agar (BA) plate and resuspended in CDM to an absorbance at 600 nm (*A*_600_) of 1.0. The inoculum was then diluted in 200 μl of CDM in a 96-well flat-bottom plate (Greiner Bio One) to a final *A*_600_ of 0.05 and sealed with gas-permeable seal (Breathe-Easy, Diversified Biotech). The plate was then incubated in a FLUOStar Omega spectrophotometer with an ACU gas controller (BMG Labtech) at 310 K in 5% CO_2_. *A*_600_ readings were recorded using the well-scan function every 30 min. Data from at least six independent growth experiments was averaged to ascertain the effect of CdCl_2_ on bacterial growth. Bacterial density at *A*_600_ was measured as the c.f.u. per ml determined by serial dilutions on BA plates.

Bacterial growth for ICP–MS, paraquat killing and glutathione assays used identical growth parameters to the microplate experiments, with MnSO_4_ and CdCl_2_ supplementation as specified. For ICP–MS, 50 ml of culture was grown to *A*_600_=0.3–0.4, harvested and prepared for analysis by ICP–MS, as described previously^23^. For the paraquat killing assays, 1 ml of *A*_600_=0.3 culture was incubated for 30 min with 10 mM paraquat and then serially diluted and plated on blood agar. Total glutathione assays were performed using 1 ml of *A*_600_=0.3 culture with the Promega GSH-Glo assay kit according to the manufacturer’s instructions (Promega, USA). To examine the effect of Cd^2+^ stress on cellular SodA activity, *S. pneumoniae* D39 was grown to an *A*_600_ of 0.3 as for the ICP–MS experiments in CDM supplemented with 1 μM MnSO_4_, 30 μM CdCl_2_:1 μM MnSO_4_ or 30 μM CdCl_2_:30 μM MnSO_4_. The cells were washed three times in PBS before lysis by sonication and intact cells and other insoluble material was removed by centrifugation at 277 K for 30 min at 14,000 *g*. The assay was performed using a SOD activity kit (Calbiochem) and the data were normalized to the total protein concentration of the cell-free extract, as determined using the Dc Bio-Rad protein determination assay, followed by normalization (in percentage) to the average SOD activity measured for the untreated cells (1 μM MnSO_4_).

Metal sensitivity assays were also performed using a *S. pneumoniae* drop test. Bacteria were grown in CDM supplemented with 1 μM MnSO_4_ and grown until the *A*_600_ reached 0.3–0.4. Cells were then serially diluted 10-fold up to a 10^−5^ dilution, and 5 μl of each dilution were spotted on BA plates supplemented with varying concentrations of ZnSO_4_ (0, 100, 300 and 1,000 μM) or CdCl_2_ (0, 10, 30 and 100 μM). Plates were photographically documented following overnight incubation at 37 °C, 5% CO_2_.

### RNA extraction and RT–PCR analysis

Pneumococci were grown as for the ICP–MS analyses, then 500 μl of the culture was mixed with 1 ml of RNA Protect (Qiagen). RNA was extracted and purified using an RNeasy Protect Bacteria Mini Kit (Qiagen) after enzymatic lysis using lysozyme and mutanolysin, all according to the manufacturer’s instructions. DNase I treatment (Roche) was performed followed by quantitative reverse transcription PCR using SuperScript III (Invitrogen) with a Roche LC480 Real-Time Cycler. The transcription levels of genes analysed were normalized to those obtained for 16S rRNA. Primer sequences are in Supplementary Table 6.

### Cell volume and concentration determination

Scanning electron microscopy was used to determine the dimensions of the pneumococcal cell. Bacteria were grown, as described above, harvested and then prepared for, and analysed by a Philips XL30 FEG scanning electron microscope as described in ref. 45. Cell measurements were obtained using instrument software. The cell dimensions were used to calculate volume assuming an ellipsoid:


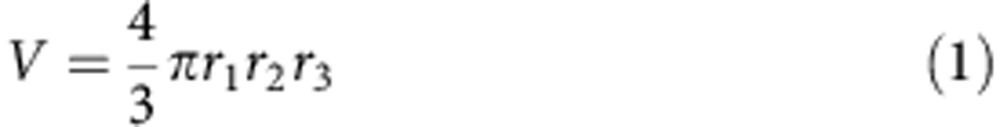


where *r*_1_, *r*_2_, and *r*_3_ are the radii of the ellipsoidal cell in three dimensions determined by scanning electron microscopy (SEM). The total quotient of transition metal ions was then derived using cell volume (*V*, litres), where the total cell density is known (c.f.u.), and concentration of metal ions in a sample of known volume and known number of cells (*M*, moles).


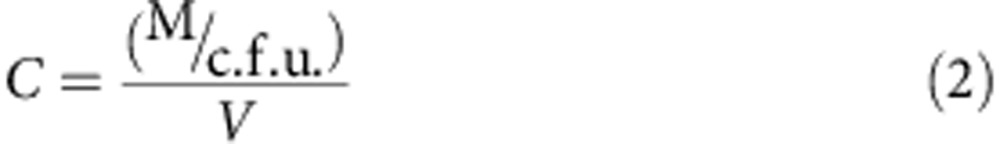


The derived concentration represents mean molarity of metal ion per cell of mean dimensions.

### Expression and purification of apo-PsaA

Recombinant PsaA was expressed in *E. coli* LEMO21(DE3) from the pCAMcLIC01-PsaA construct^8^. The dodecahistidine tag was removed from affinity-purified PsaA by enzymatic digestion by the human rhinovirus 3C protease and the protein purified further on a HisTrap HP column. Apo-PsaA was prepared by dialyzing the dodecahistidine tag-cleaved protein in a 20-kDa molecular-weight-cutoff membrane (Slide-A-Lyzer, Pierce) against 4 l of sodium acetate buffer, pH 4.0, with 20 mM EDTA at 300 K. The sample was then dialysed against 4 l of 20 mM Tris-HCl, pH 7.2, and 100 mM NaCl, at 277 K and centrifuged at 18,000 *g* for 10 min to remove any insoluble material. Protein samples were analysed for metal content by heating 5 μM protein at 370 K for 15 min in 3.5% HNO_3_ and the metal ion content was measured by ICP–MS.

### Protein assays

Protein concentration determination was performed using the Dc Bio-Rad protein determination assay. Metal-loading assays were performed on purified apo-PsaA (30 μM) by mixing with 300 μM CdCl_2_ in a total volume of 2 ml in the assay buffer (20 mM MOPS, pH 7.2, and 100 mM NaCl) for 60 min at 277 K. The sample was desalted on a PD10 column (GE Healthcare) into the assay buffer and the protein concentration was quantified. Protein was then either kept for ICP–MS analysis or mixed with 3 mM EDTA in a total volume of 2 ml for 60 min at 277 K. Samples were then desalted on a PD10 column as before. Solutions (5–10 μM) of control, metal-loaded and EDTA-treated protein were prepared in 3.5% HNO_3_ and boiled for 15 min at 370 K. Samples were then cooled and centrifuged for 20 min at 14,000 *g*. The supernatant was then analysed by ICP–MS and the protein-to-metal ratio was determined.

### Immunoblot analyses of PsaA expression levels

Wild-type and mutant *S. pneumoniae* were grown under the same conditions as for ICP–MS. After reaching an *A*_600_ of 0.4, cells were incubated with 0.1% sodium deoxycholate at 310 K for 60 min to induce lysis. Protein concentrations were determined and 10 μg of total protein was loaded into each lane. After electrophoretic separation by SDS–PAGE, proteins were transferred to a nitrocellulose membrane using the iBlot (Life Technologies) system. The blots were incubated with murine anti-PsaA serum (1:2,000; ref. 23), followed by anti-mouse IRDye 800 (LI-COR; 1:50,000), and were scanned using an Odyssey infrared imaging system (LI-COR). Band intensities were measured using the manufacturer’s application software and the results correspond to the mean (±s.e.m.) of two independent biological experiments.

### Determination of *K*_D_ for PsaA with Cd^2+^

Excitation–emission spectra were determined on a FLUOStar Omega (BMG Labtech) at 301 K using black half-volume 384-well microtitre plates (Greiner Bio One). All experiments were performed in 20 mM MOPS (pH 6.7) and 50 mM NaCl with FluoZin-3 (Life Technologies) at a concentration of 50 nM. Deionized water and buffers were prepared and treated with Chelex-100 (Sigma-Aldrich) to avoid metal contamination. Filters used for FluoZin-3 were excitation (485±10 nm) and emission (520±5 nm). To determine the dissociation constant between a metal (X) and FluoZin-3 (F), we considered the following equilibria:


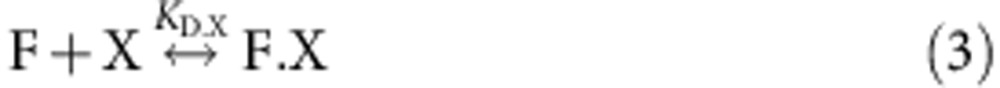


where, for a metal that increases the fluorescence of the probe by more than ~10%, the following equation, which is an exact analytical relationship derived from the mass action equation for the formation of a 1:1 complex between probe and metal, was used to estimate the dissociation constant, *K*_D.X_


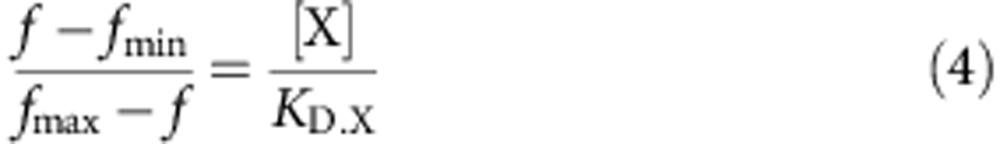


where *f* is the measured fluorescence intensity in the presence of metal, *f*_max_ is the fluorescence in the presence of saturating metal and *f*_min_ is the fluorescence in the absence of metal. In all cases, a low concentration (50 nM) of probe was used and we assumed that the free-metal concentration was equal to the added metal concentration. The mean±s.e.m. (*n*=6) *K*_D_ determined for FluoZin-3 with CdCl_2_ in the buffer system used in this study was determined to be 250±35 nM. Competition by PsaA for Cd^2+^ binding was assessed by monitoring the decrease in the fluorescence of 50 nM FluoZin-3-Cd^2+^ in response to increasing apo-PsaA concentrations and analysed using log_10_[inhibitor] versus response model, with the experimentally derived *K*_D_ for FluoZin-3 with Cd^2+^, in GraphPad prism to determine the *K*_D_ value for Cd^2+^ binding by PsaA.

### Protein crystallization and structure determination

Cd-bound PsaA-D280N protein crystals were obtained in 12.5% (w/v) polyethylene glycol (PEG) 1000, 12.5% (w/v) PEG 3350, 12.5% (v/v) MPD, 0.1 M Trizma-Bicine, pH 8.7, and 0.01 M CdCl_2_ using the hanging drop vapour diffusion method^8^. Cd^2+^-bound wild-type PsaA crystals were grown in 26–36% PEG 400, 0.1 M NaCl, 0.1 M Trizma-HCl, pH 8.0, and 0.1 M CdCl_2_ also using vapour diffusion. Before data collection, the crystals were flash-cooled by rapid immersion in liquid nitrogen. The diffraction data were collected on a single crystal at the Australian Synchrotron (MX2 beamline). Cd^2+^-bound D280N and wild-type PsaA structures were determined by molecular replacement with Phaser^46^ using the Mn^2+^-bound D280N (PDB accession code 3ZKA) and the Zn^2+^-bound (PDB accession code 1PSZ) PsaA crystal structures, respectively, as phasing models followed by automatic model building in Phenix.AutoBuild^47^. The structures were refined iteratively using Phenix.Refine and Refmac5 manual model fitting in Coot^48^. Both structures had two molecules (chains A and B) in each asymmetric unit and chain A molecules were used in subsequent structural analyses. Structural analyses (superpositions, metal ion coordination and N-/C-terminal domain-crossing angles) were performed in Chimera^49^. Secondary structure elements were assigned by DSSP (version 2.1.0; ref. 50). The WHAT-IF^51^ and PISA^52^ web services were used to identify hydrogen bonds and salt bridges. Data processing and structure refinement statistics can be found in Supplementary Table 7.

## Author contributions

S.L.B., B.A.E. and C.A.M. designed and executed all the biochemical studies. C.-l.Y.O. generated the mutant strains. Z.L., R.M.C. and M.J.M. designed and executed the crystallographic experiments. S.L.B., R.M.C., M.J.M., M.L.O. and C.A.M. drafted the manuscript. All authors contributed to the design, analysis, discussion of the research and writing of the final manuscript.

## Additional information

**Accession codes:** Coordinates and structure factors for Cd^2+^-bound PsaA-D280N, partially occluded state, and for Cd^2+^-bound wild-type PsaA, closed state, have been deposited in the RCSB Protein Data Bank under accession codes 4uto and 4utp, respectively.

**How to cite this article:** Begg, S. L. *et al*. Dysregulation of transition metal ion homeostasis is the molecular basis for cadmium toxicity in *Streptococcus pneumoniae*. *Nat. Commun.* 6:6418 doi: 10.1038/ncomms7418 (2015).

## Supplementary Material

Supplementary Information Supplementary Figures 1-8 and Supplementary Tables 1-7

## Figures and Tables

**Table 1:** *S. pneumoniae* cell volume parameters.

**Strain**	Cell volume (fl)^*^
D39 1 μM Mn^2+^	0.113±0.04
D39 1 μM Mn^2+^: 30 μM Cd^2+^	0.124±0.07
D39 30 μM Mn^2+^: 30 μM Cd^2+^	0.127±0.07

^*^The data correspond to the mean±s.d. from >40 independent measurements.

## References

[b1] Nriagu J. O. & Pacyna J. M. Quantitative assessment of worldwide contamination of air, water and soils by trace metals. Nature 333, 134–139 (1988). 3285219 10.1038/333134a0

[b2] Joseph P. Mechanisms of cadmium carcinogenesis. Toxicol. Appl. Pharmacol. 238, 272–279 (2009). 19371617 10.1016/j.taap.2009.01.011

[b3] Lane T. W. . A cadmium enzyme from a marine diatom. Nature 435, 42 (2005). 15875011 10.1038/435042a

[b4] Cuypers A. . Cadmium stress: an oxidative challenge. Biometals 23, 927–940 (2010). 20361350 10.1007/s10534-010-9329-x

[b5] Archibald F. S. & Duong M. N. Manganese acquisition by *Lactobacillus plantarum*. J. Bacteriol. 158, 1–8 (1984). 6715278 10.1128/jb.158.1.1-8.1984 PMC215370

[b6] Hubert N. & Hentze M. W. Previously uncharacterized isoforms of divalent metal transporter (DMT)-1: implications for regulation and cellular function. Proc. Natl Acad. Sci. USA 99, 12345–12350 (2002). 12209011 10.1073/pnas.192423399 PMC129447

[b7] McAllister L. J. . Molecular analysis of the *psa* permease complex of *Streptococcus pneumoniae*. Mol. Microbiol. 53, 889–901 (2004). 15255900 10.1111/j.1365-2958.2004.04164.x

[b8] Couñago R. M. . Imperfect coordination chemistry facilitates metal ion release in the Psa permease. Nat. Chem. Biol. 10, 35–41 (2014). 24212134 10.1038/nchembio.1382

[b9] Dintilhac A. & Claverys J. P. The *adc* locus, which affects competence for genetic transformation in *Streptococcus pneumoniae*, encodes an ABC transporter with a putative lipoprotein homologous to a family of streptococcal adhesins. Res. Microbiol. 148, 119–131 (1997). 9765793 10.1016/S0923-2508(97)87643-7

[b10] Outten C. E. & O'Halloran T. V. Femtomolar sensitivity of metalloregulatory proteins controlling zinc homeostasis. Science 292, 2488–2492 (2001). 11397910 10.1126/science.1060331

[b11] Irving H. & Williams R. J. P. The stability of transition-metal complexes. J. Chem. Soc. 3192–3210 (1953).

[b12] Lisher J. P., Higgins K. A., Maroney M. J. & Giedroc D. P. Physical characterization of the manganese-sensing pneumococcal surface antigen repressor from *Streptococcus pneumoniae*. Biochemistry 52, 7689–7701 (2013). 24067066 10.1021/bi401132w PMC3859839

[b13] Plumptre C. D. . AdcA and AdcAII employ distinct zinc acquisition mechanisms and contribute additively to zinc homeostasis in *Streptococcus pneumoniae*. Mol. Microbiol. 91, 834–851 (2014). 24428621 10.1111/mmi.12504

[b14] Dintilhac A., Alloing G., Granadel C. & Claverys J. P. Competence and virulence of *Streptococcus pneumoniae*: Adc and PsaA mutants exhibit a requirement for Zn and Mn resulting from inactivation of putative ABC metal permeases. Mol. Microbiol. 25, 727–739 (1997). 9379902 10.1046/j.1365-2958.1997.5111879.x

[b15] Reyes-Caballero H. . The metalloregulatory zinc site in *Streptococcus pneumoniae* AdcR, a zinc-activated MarR family repressor. J. Mol. Biol. 403, 197–216 (2010). 20804771 10.1016/j.jmb.2010.08.030 PMC2949468

[b16] Loisel E. . AdcAII, a new pneumococcal Zn-binding protein homologous with ABC transporters: biochemical and structural analysis. J. Mol. Biol. 381, 594–606 (2008). 18632116 10.1016/j.jmb.2008.05.068

[b17] Kloosterman T. G., van der Kooi-Pol M. M., Bijlsma J. J. & Kuipers O. P. The novel transcriptional regulator SczA mediates protection against Zn^2+^ stress by activation of the Zn^2+^-resistance gene *czcD* in *Streptococcus pneumoniae*. Mol. Microbiol. 65, 1049–1063 (2007). 17640279 10.1111/j.1365-2958.2007.05849.x

[b18] Anton A., Grosse C., Reissmann J., Pribyl T. & Nies D. H. CzcD is a heavy metal ion transporter involved in regulation of heavy metal resistance in *Ralstonia* sp. strain CH34. J. Bacteriol. 181, 6876–6881 (1999). 10559151 10.1128/jb.181.22.6876-6881.1999 PMC94160

[b19] Rosch J. W., Gao G., Ridout G., Wang Y. D. & Tuomanen E. I. Role of the manganese efflux system *mntE* for signalling and pathogenesis in *Streptococcus pneumoniae*. Mol. Microbiol. 72, 12–25 (2009). 19226324 10.1111/j.1365-2958.2009.06638.x PMC2706702

[b20] Crupper S. S., Worrell V., Stewart G. C. & Iandolo J. J. Cloning and expression of *cadD*, a new cadmium resistance gene of *Staphylococcus aureus*. J. Bacteriol. 181, 4071–4075 (1999). 10383976 10.1128/jb.181.13.4071-4075.1999 PMC93898

[b21] Fu Y. . A new structural paradigm in copper resistance in *Streptococcus pneumoniae*. Nat. Chem. Biol. 9, 177–183 (2013). 23354287 10.1038/nchembio.1168 PMC3578076

[b22] Brenot A., Weston B. F. & Caparon M. G. A PerR-regulated metal transporter (PmtA) is an interface between oxidative stress and metal homeostasis in *Streptococcus pyogenes*. Mol. Microbiol. 63, 1185–1196 (2007). 17238923 10.1111/j.1365-2958.2006.05577.x

[b23] McDevitt C. A. . A molecular mechanism for bacterial susceptibility to zinc. PLoS Pathog. 7, e1002357 (2011). 22072971 10.1371/journal.ppat.1002357 PMC3207923

[b24] Lawrence M. C. . The crystal structure of pneumococcal surface antigen PsaA reveals a metal-binding site and a novel structure for a putative ABC-type binding protein. Structure 6, 1553–1561 (1998). 9862808 10.1016/s0969-2126(98)00153-1

[b25] Braun W. . Comparison of the NMR solution structure and the x-ray crystal structure of rat metallothionein-2. Proc. Natl Acad. Sci. USA 89, 10124–10128 (1992). 1438200 10.1073/pnas.89.21.10124 PMC50290

[b26] Graham S. C., Maher M. J., Simmons W. H., Freeman H. C. & Guss J. M. Structure of *Escherichia coli* aminopeptidase P in complex with the inhibitor apstatin. Acta Crystallogr. D Biol. Crystallogr. 60, 1770–1779 (2004). 15388923 10.1107/S0907444904018724

[b27] Price N. M. & Morel F. M. M. Cadmium and cobalt substitution for zinc in a marine diatom. Nature 344, 658–660 (1990).

[b28] Xu Y., Feng L., Jeffrey P. D., Shi Y. & Morel F. M. Structure and metal exchange in the cadmium carbonic anhydrase of marine diatoms. Nature 452, 56–61 (2008). 18322527 10.1038/nature06636

[b29] Ogunniyi A. D. . Central role of manganese in regulation of stress responses, physiology, and metabolism in *Streptococcus pneumoniae*. J. Bacteriol. 192, 4489–4497 (2010). 20601473 10.1128/JB.00064-10 PMC2937371

[b30] Eijkelkamp B. A. . Extracellular zinc competitively inhibits manganese uptake and compromises oxidative stress management in *Streptococcus pneumoniae*. PLoS ONE 9, e89427 (2014). 24558498 10.1371/journal.pone.0089427 PMC3928430

[b31] Yesilkaya H. . Role of manganese-containing superoxide dismutase in oxidative stress and virulence of *Streptococcus pneumoniae*. Infect. Immun. 68, 2819–2826 (2000). 10768978 10.1128/iai.68.5.2819-2826.2000 PMC97493

[b32] Potter A. J., Trappetti C. & Paton J. C. *Streptococcus pneumoniae* uses glutathione to defend against oxidative stress and metal ion toxicity. J. Bacteriol. 194, 6248–6254 (2012). 22984260 10.1128/JB.01393-12 PMC3486410

[b33] Tseng H. J., McEwan A. G., Paton J. C. & Jennings M. P. Virulence of *Streptococcus pneumoniae*: PsaA mutants are hypersensitive to oxidative stress. Infect. Immun. 70, 1635–1639 (2002). 11854257 10.1128/IAI.70.3.1635-1639.2002 PMC127802

[b34] Hider R. C. & Kong X. L. Glutathione: a key component of the cytoplasmic labile iron pool. Biometals 24, 1179–1187 (2011). 21769609 10.1007/s10534-011-9476-8

[b35] Tabares L. C. & Un S. *In situ* determination of manganese(II) speciation in *Deinococcus radiodurans* by high magnetic field EPR: detection of high levels of Mn(II) bound to proteins. J. Biol. Chem. 288, 5050–5055 (2013). 23303180 10.1074/jbc.C112.444992 PMC3576107

[b36] Sun X. . Putative copper- and zinc-binding motifs in *Streptococcus pneumoniae* identified by immobilized metal affinity chromatography and mass spectrometry. Proteomics 11, 3288–3298 (2011). 21751346 10.1002/pmic.201000396

[b37] Andreini C., Bertini I., Cavallaro G., Holliday G. L. & Thornton J. M. Metal ions in biological catalysis: from enzyme databases to general principles. J. Biol. Inorg. Chem. 13, 1205–1218 (2008). 18604568 10.1007/s00775-008-0404-5

[b38] Ralston D. M. & O'Halloran T. V. Ultrasensitivity and heavy-metal selectivity of the allosterically modulated MerR transcription complex. Proc. Natl Acad. Sci. USA 87, 3846–3850 (1990). 2187194 10.1073/pnas.87.10.3846 PMC54000

[b39] Guerra A. J. & Giedroc D. P. Metal site occupancy and allosteric switching in bacterial metal sensor proteins. Arch. Biochem. Biophys. 519, 210–222 (2012). 22178748 10.1016/j.abb.2011.11.021 PMC3312040

[b40] Sun Y., Wong M. D. & Rosen B. P. Role of cysteinyl residues in sensing Pb(II), Cd(II), and Zn(II) by the plasmid pI258 CadC repressor. J. Biol. Chem. 276, 14955–14960 (2001). 11278706 10.1074/jbc.M010595200

[b41] Ma Z. . Bacillithiol is a major buffer of the labile zinc pool in *Bacillus subtilis*. Mol. Microbiol. 94, 756–770 (2014). 25213752 10.1111/mmi.12794 PMC4227968

[b42] Ong C. L., Gillen C. M., Barnett T. C., Walker M. J. & McEwan A. G. An antimicrobial role for zinc in innate immune defense against group A *Streptococcus*. J. Infect. Dis. 209, 1500–1508 (2014). 24449444 10.1093/infdis/jiu053

[b43] Helbig K., Bleuel C., Krauss G. J. & Nies D. H. Glutathione and transition-metal homeostasis in *Escherichia coli*. J. Bacteriol. 190, 5431–5438 (2008). 18539744 10.1128/JB.00271-08 PMC2493246

[b44] Rajkarnikar A. . Analysis of mutants disrupted in bacillithiol metabolism in *Staphylococcus aureus*. Biochem. Biophys. Res. Commun. 436, 128–133 (2013). 23618856 10.1016/j.bbrc.2013.04.027 PMC3960916

[b45] Fairbrother L. . Effect of the cyanide-producing bacterium *Chromobacterium violaceum* on ultraflat Au surfaces. Chem. Geol. 265, 313–320 (2009).

[b46] McCoy A. J. Solving structures of protein complexes by molecular replacement with Phaser. Acta Crystallogr. D Biol. Crystallogr. 63, 32–41 (2007). 17164524 10.1107/S0907444906045975 PMC2483468

[b47] Terwilliger T. C. . Iterative model building, structure refinement and density modification with the PHENIX AutoBuild wizard. Acta Crystallogr. D Biol. Crystallogr. 64, 61–69 (2008). 18094468 10.1107/S090744490705024X PMC2394820

[b48] Emsley P., Lohkamp B., Scott W. G. & Cowtan K. Features and development of Coot. Acta Crystallogr. D Biol. Crystallogr. 66, 486–501 (2010). 20383002 10.1107/S0907444910007493 PMC2852313

[b49] Pettersen E. F. . UCSF Chimera--a visualization system for exploratory research and analysis. J. Comput. Chem. 25, 1605–1612 (2004). 15264254 10.1002/jcc.20084

[b50] Kabsch W. & Sander C. Dictionary of protein secondary structure: pattern recognition of hydrogen-bonded and geometrical features. Biopolymers 22, 2577–2637 (1983). 6667333 10.1002/bip.360221211

[b51] Hekkelman M. L. . WIWS: a protein structure bioinformatics Web service collection. Nucleic Acids Res. 38, W719–W723 (2010). 20501602 10.1093/nar/gkq453 PMC2896166

[b52] Krissinel E. & Henrick K. Inference of macromolecular assemblies from crystalline state. J. Mol. Biol. 372, 774–797 (2007). 17681537 10.1016/j.jmb.2007.05.022

