# Supplementary material for: Dysregulation of transition metal ion homeostasis is the molecular basis for cadmium toxicity in Streptococcus pneumoniae
Source: Nat Commun. 2015 Mar 3;6:6418. doi: 10.1038/ncomms7418 (PMC4366526; doi:10.1038/ncomms7418)
Supplement: Supplementary Information — Supplementary Figures 1-8 and Supplementary Tables 1-7 [file ncomms7418-s1.pdf]

Supplementary Figure. 1

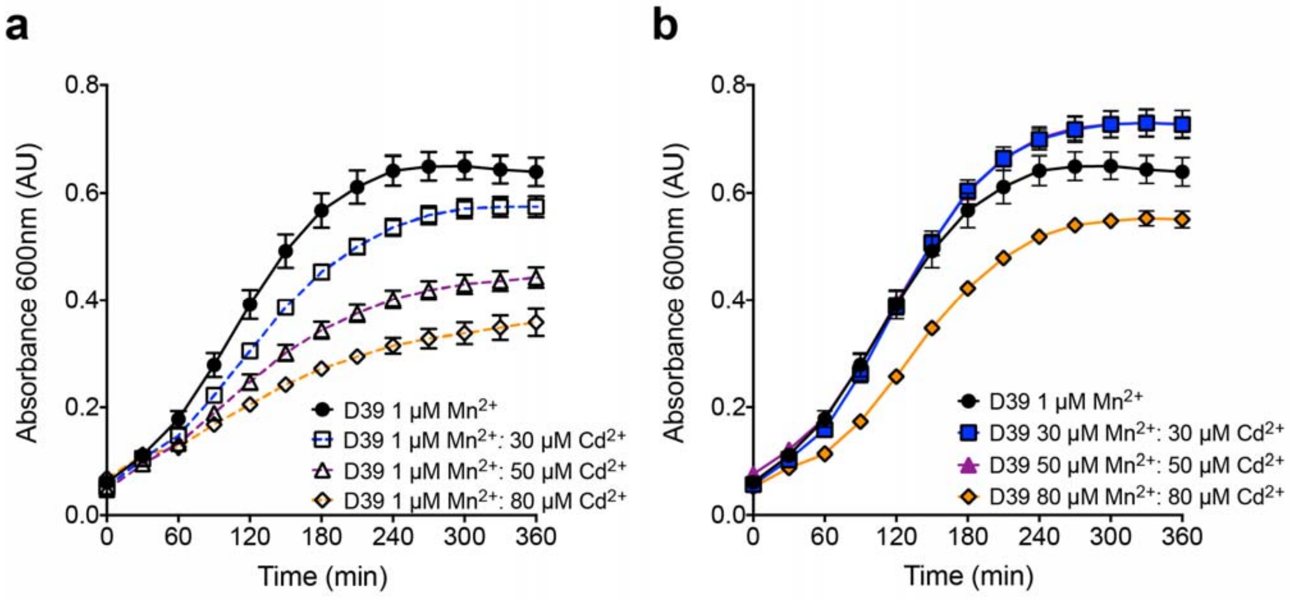

Supplementary Figure. 1. The effect of Cd^{2+} on *S. pneumoniae* growth. (**a** and **b**) *S. pneumoniae* (D39) grown in CDM supplemented with metal ions as indicated. The data correspond to mean (± s.e.m.) absorbance at 600 nm measurements from three independent biological experiments. Errors bars, where not visible, are overlapped by the representative symbols. Data is plotted on a linear scale to aid visualization.

Supplementary Figure 2.

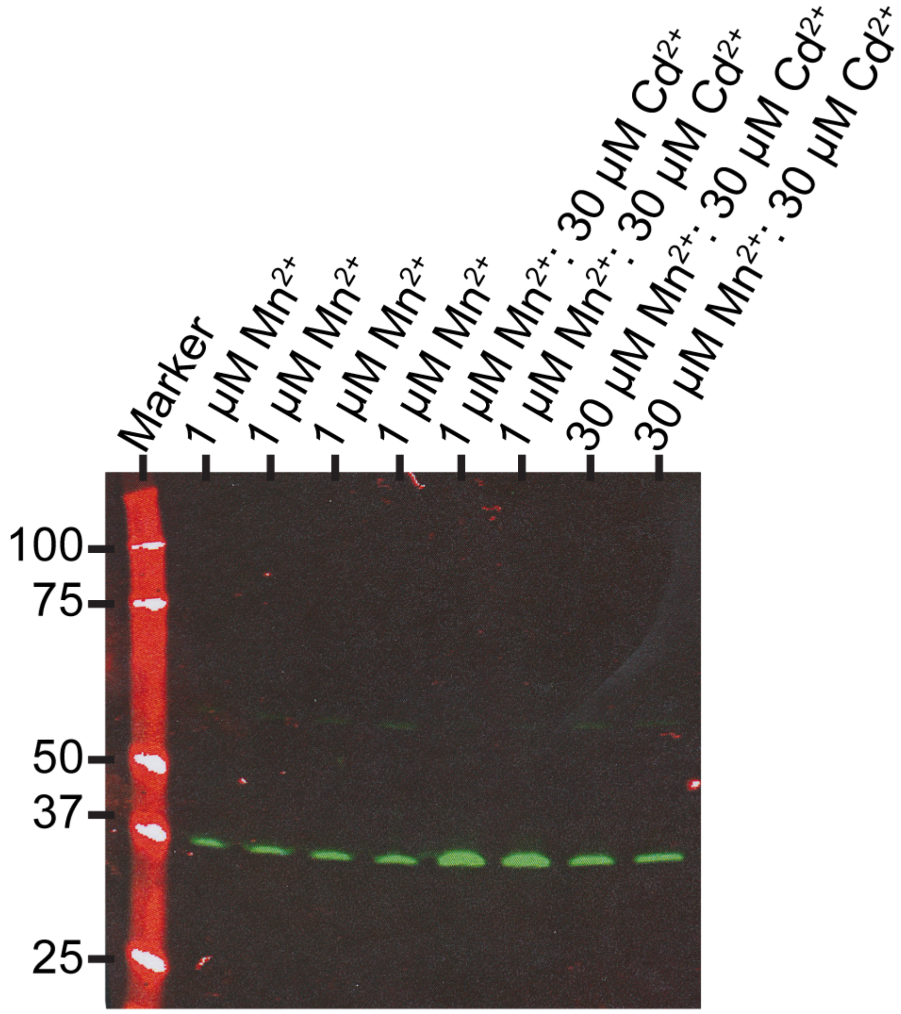

Supplementary Figure 2. PsaA quantitation. Representative, uncropped immunoblot for the analysis of the relative amounts of PsaA protein expression shown in Figure 1e. Equal amounts of total protein (10 μg) from whole-cell extracts of *S. pneumoniae* grown in CDM supplemented with 1 μM Mn^{2+}, 1 μM Mn^{2+}: 30 μM Cd^{2+}, and 30 μM Mn^{2+}: 30 μM Cd^{2+} were loaded in each lane, as shown. PsaA was detected using polyclonal murine antiserum (1:2,000) followed by an infrared dye labelled secondary antibody (1:50,000). The membrane was scanned using an Odyssey imaging system (LI-COR Biosciences).

Supplementary Figure 3.

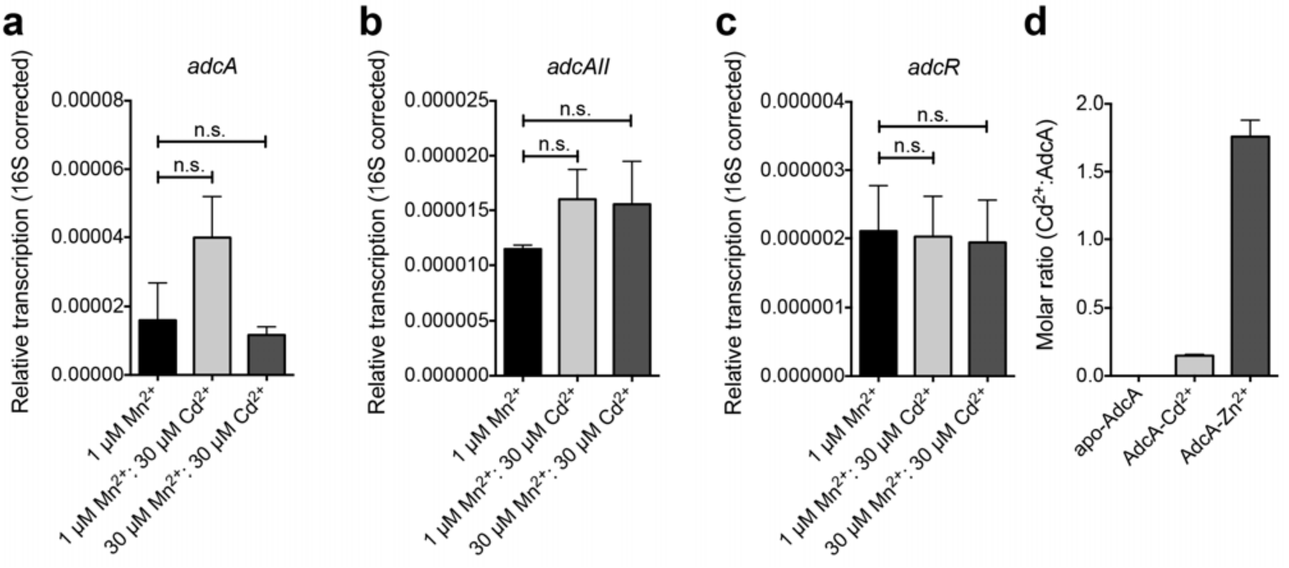

Supplementary Figure 3. The effect of Cd^{2+} on the regulation of the Zn^{2+}-acquisition pathway. Relative transcription, corrected to 16S rRNA, of *adcA* (**a**), *adcAII* (**b**), and *adcR* (**c**) by *S. pneumoniae* when grown in CDM supplemented with 1 µM Mn^{2+} (black), 1 µM Mn^{2+}: 30 µM Cd^{2+} (light grey), and 30 µM Mn^{2+}: 30 µM Cd^{2+} (dark grey). The data correspond to the mean (± s.e.m.) of three independent biological experiments. The statistical significance of the differences in the mean data was determined by two-tailed unpaired *t*-tests (n.s. corresponds to 'not significant'). (**d**) *In vitro* metal binding of Cd^{2+} and Zn^{2+} to apo-AdcA analysed by ICP-MS. Data corresponds to the mean (± s.d.) molar ratio of Cd^{2+} or Zn^{2+} to AdcA for apo-AdcA (black), Cd^{2+}-AdcA (light grey), Zn^{2+}-AdcA (dark grey).

Supplementary Figure 4.

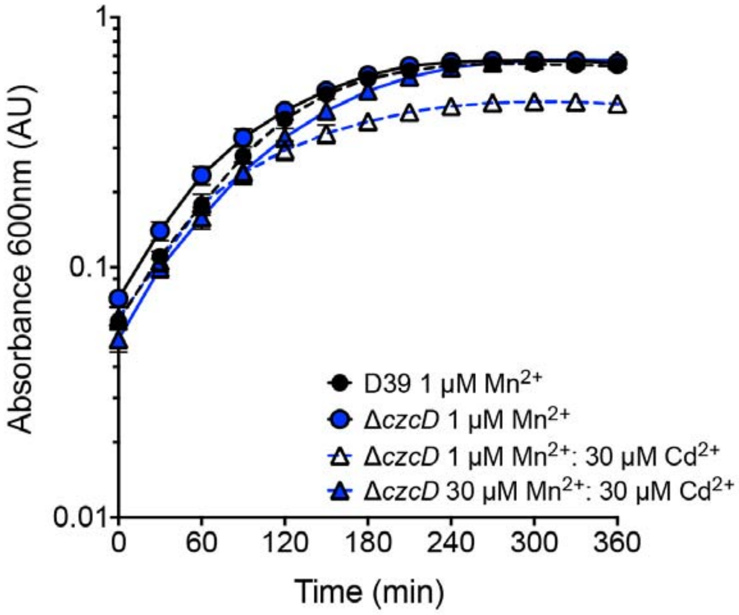

Supplementary Figure 4. The impact of Cd^{2+} on *S. pneumoniae* Δ*czcD*. *S. pneumoniae* wild-type (D39) and Δ*czcD* grown in CDM supplemented with metal ions as indicated. The data correspond to the mean (± s.e.m.) absorbance 600 nm measurements from three independent biological experiments. Errors bars, where not visible, are overlapped by the representative symbols.

Supplementary Figure 5.

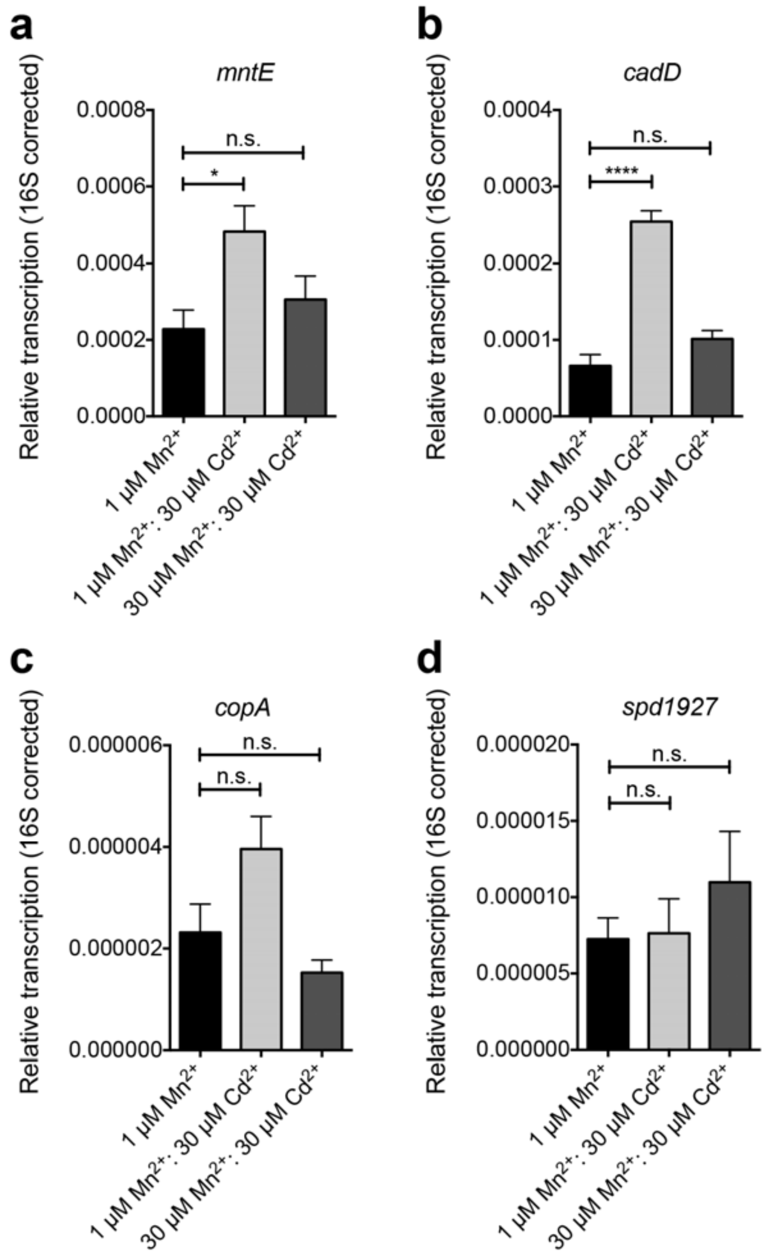

Supplementary Figure 5. The effect of Cd^{2+} on known and putative metal ion transporters. Relative transcription, corrected to 16S rRNA, of *mntE* (**a**), *cadD* (**b**), *copA* (**c**), and *spd1927* (**d**). The data correspond to the mean (± s.e.m.), of three independent biological experiments, by *S. pneumoniae* when grown in CDM supplemented with 1 µM Mn^{2+} (black), 30 µM Cd^{2+}: 1 µM Mn^{2+} (light grey), and 30 µM Cd^{2+}: 30 µM Mn^{2+} (dark grey). The statistical significance of the differences in the mean data was determined by two-tailed unpaired *t*-tests (n.s. corresponds to 'not significant', * corresponds to $P < 0.05$, and **** to $P < 0.0001$).

Supplementary Figure 6.

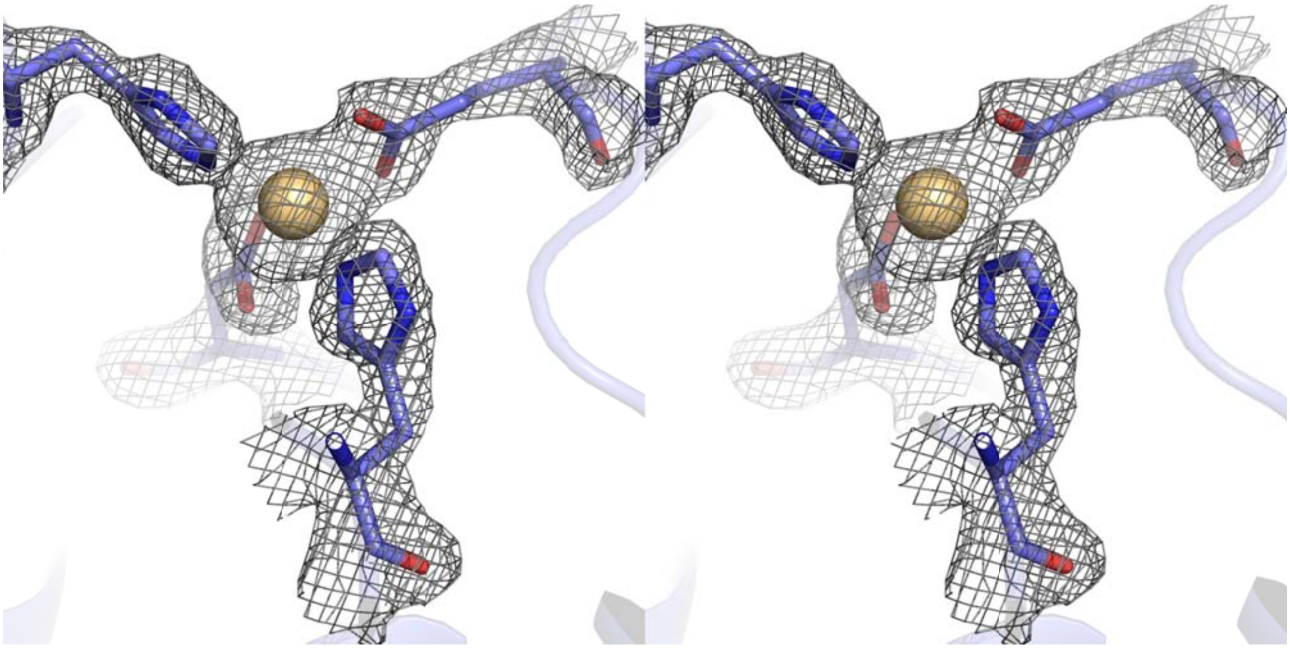

Supplementary Figure 6. Stereo cartoon representation of the metal binding site in PsaA-Cd^{2+}. Electron density (2Fo-Fc) for the bound Cd ion (gold sphere) and coordinating residues (sticks) is shown as a grey mesh, contoured at 1.6 σ.

Supplementary Figure 7.

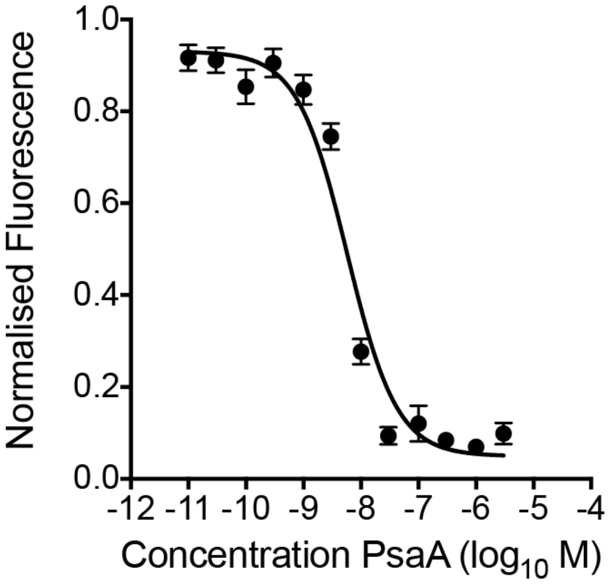

Supplementary Figure 7. Competitive Cd^{2+} binding by apo-PsaA from FluoZin-3-Cd^{2+}. Apo-PsaA was titrated against 50 nM FluoZin-3-Cd^{2+} until fluorescence was quenched. The fluorescence data was normalized using experimentally observed fluorescence minimum and maximum values. Each data point corresponds to the mean (± s.e.m.) of three independent experiments.

Supplementary Figure 8.

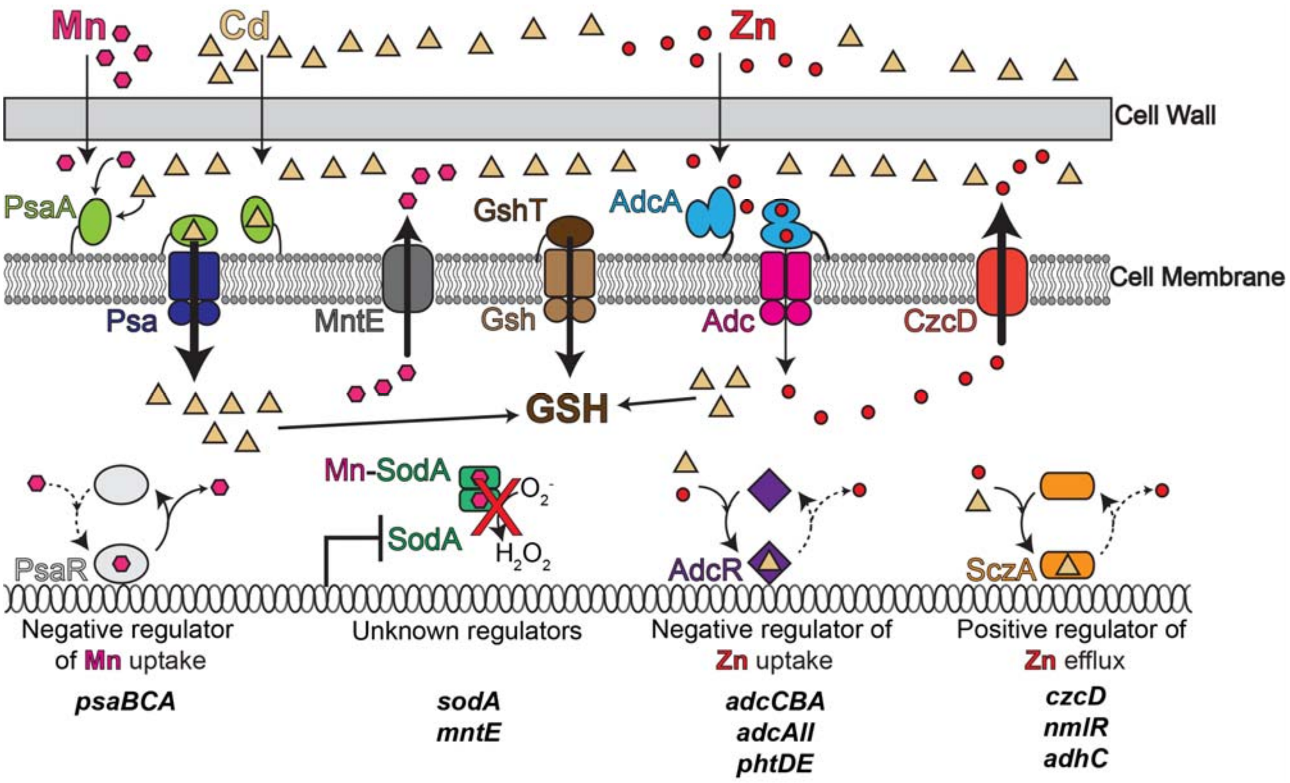

Supplementary Figure 8. Model for Cd^{2+} dysregulation of transition metal ion homeostasis. In the presence of extracellular Cd^{2+} exposure, Cd^{2+} competes with Mn^{2+} for PsaA for uptake via the Psa permease. Cadmium entry into the cell also up-regulates MntE expression, exacerbating the Mn^{2+} depletion. PsaR, the negative regulator of PsaBCA, is de-repressed and drives increased transcription of the Psa permease and, thereby, imports more Cd^{2+}. Depletion of Mn^{2+} also down-regulates *sodA* transcription via an unknown regulator. Accumulation of Cd^{2+} dysregulates Zn^{2+} homeostasis by repressing AdcA and AdcAII expression, most likely via the negative regulator AdcR. Cadmium also activates CzcD expression, presumably via mismetallation of the positive regulator SczA. The concerted action of these regulatory processes is to drive depletion of cellular Zn^{2+}. Glutathione acquisition, via the Gsh permease, allows Cd^{2+} to be buffered intracellularly, but when the concentration of Cd^{2+} exceeds that of glutathione, Cd^{2+} can mismetallate cellular proteins. Solid lines represent a process that is occurring, e.g. metal binding and activation of a transcriptional regulator. Dotted lines represent the corresponding opposing response that is not occurring, e.g. the dissociation of a metal ion from a regulator.

Supplementary Tables

Supplementary Table 1.

***S. pneumoniae* D39 and mutant variants Mn^{2+}, Zn^{2+} and Cd^{2+} accumulation**

Strain	Mn^{2+} (mM) [a]	Zn^{2+} (mM)	Cd^{2+} (mM)
D39 1 μM Mn^{2+}	8.60 ± 0.50	6.82 ± 0.33	0.01 ± 0.01
D39 1 μM Mn^{2+}: 30 μM Cd^{2+}	0.78 ± 0.07	2.58 ± 0.43	46.41 ± 2.93
D39 30 μM Mn^{2+}: 30 μM Cd^{2+}	5.20 ± 0.21	3.53 ± 0.37	16.47 ± 0.67

[a] The data correspond to the mean ± s.e.m. from 3 independent biological experiments.

Supplementary Table 2. Comparison of metal-protein distances for coordinating atoms in the metal-binding site of PsaA

Coordinator	Distance Cd-PsaA (Å)	Distance Zn-PsaA (Å)	Distance Mn-PsaA (Å)
His 67 Nε2	2.3	2.0	2.1
His 139 Nε2	2.3	2.0	2.2
Glu 205 Oε1	2.4	2.1	2.1
Glu 205 Oε2	2.5	2.6 [a]	2.4
Asp 280 Oδ1	2.7 [a]	2.8 [a]	2.4
Asp 280 Oδ2	2.1	2.0	2.1

[a] Outside the cut-off limit of 2.5 and 2.7 Å for bidentate coordination to Zn and Cd, respectively.

Supplementary Table 3. Coordination number and geometry for various metal ions in the metal-binding site of PsaA

PsaA-Metal (PDB Accession code)	Coordination Number	Geometry	Angle RMSD (ideal; degrees)
Cd-PsaA (4utp)	5	trigonal-bipyramidal / square-pyramidal	18.73 / 16.74
Zn-PsaA (1psz)	4	tetrahedral	13.07
Mn-PsaA (3ztt)	6	octahedral / trigonal-prism	20.70 / 18.97

Supplementary Table 4. Coordination numbers and geometries of spurious, mixed N/O Cd^{2+} sites.

Geometry[a]	Coordination number	Frequency (n = 161)
Octahedron	6	39.8 %
Square pyramid	5	18.6 %
Pentagonal bipyramid	7	16.1 %
Tetrahedron	4	7.5 %
Square planar	4	6.8 %
Trigonal bipyramid	5	3.1 %

[a] Protein Cd^{2+}-binding sites identified using MetalPDB (http://metalweb.cerm.unifi.it), May 2014.

Supplementary Table 5. Domain movement and distortion of the flexible region in the linking helix of various PsaA structures.

Protein	Main-chain H-bonds (184-194) [a]	Hinge / closing angle (^{o}) [b]
Open Apo-PsaA (3zk7)	12	63.9 / 0.0
Open Cd-PsaA (4uto)	11	62.9 / 1.0
Closed Cd-PsaA (4utp)	8	52.9 / 11.0
Open Mn-PsaA (3zka)	11	60.6 / 3.3
Closed Mn-PsaA (3ztt)	8	53.6 / 10.3
Closed Zn-PsaA (1psz)	7	52.1 / 11.8

[a] Hydrogen bonds identified by WHAT-IF [48] and confirmed visually in PyMOL (Version 1.3 Schrödinger, LLC).

[b] Hinge angles were calculated in Chimera [46] following superposition of residues 32-163 from indicated PsaA structures onto the equivalent region of metal-free, open PsaA. The hinge angle was defined here as the angle between an axis defined by backbone atoms from residues 167-192 in the linking helix of the metal-free, open PsaA structure (PDB accession code 3zk7) and a second axis defined by backbone atoms from residues 233-245 in the helix H8 of various PsaA structures. The closing angle was defined here as the difference between the hinge angles of the open, metal-free PsaA structure and that of the indicated structures.

Supplementary Table 6. Oligonucleotide sequences

Primer name	Sequence 5' - 3'
RT-qPCR Primers	
qSPD0016_F	CATGCAAGTAGAACGCTGAA
qSPD0016_R	TGTCATGCAACATCCACTCT
qSPD0635_F	CGTCTTGCCAGAAGAAAAAG
qSPD0635_R	GACTCGATGGCGATATCTGT
qSPD0667_F	ACACTTGAACCACGCTCTTT
qSPD0667_R	CAACCTGAACCAAAACGAGT
qSPD1383_F	CGATTGCTTACCCAGTTCAT
qSPD1383_R	GCCAACTGTCAAGATGGATT
qSPD1384_F	TCAAGTTAGCTGAGCGTGGA
qSPD1384_R	TAAGAGGGCCACATTTCCAA
qSPD1436_F	TCCCAGTAAAACCAGCCAAC
qSPD1436_R	GTTTCCTAGCCAGTGGGTCA
qSPD1438_F	TATGCGGGGCAATATCTAGG
qSPD1438_R	AACAATTGCAAAGCGAATCC
qSPD1463_F	AGCCTATGGTGTTCCAAGTG
qSPD1463_R	GTTTTCATTGGACGGTCATC
qSPD1638_F	TTGGTTCTAGCGCTGTTCTT
qSPD1638_R	AGGCTCCTAGCAGGCTAAAC
qSPD1927_F	GGAGAGTTACGCATTCGTGT
qSPD1927_R	CCCAGACCCCAAGAAATTAT
Mutagenesis Primers	
SPD1384_KO_F1	GCGATATCTTCCCCACCAGA

SPD1384_KO_R1	TTGTTCATGTAATCACTCCTTCTCCACGCTCAGCTAACTTGA
SPD1384_KO_F2	CGGGAGGAAATAATTCTATGAGCCACGAAACCTGGCAAAATATC
SPD1384_KO_R2	ACGAGCCAACCAATGACCTA
SPD0150_KO_F1	TCCACAACCTCAAAACGGTG
SPD0150_KO_R1	CGTATGTATTCAAATATATCCTCCTCAAGTCCTAGGGCAGCAAGAG
SPD0150_KO_F2	TAACTATAAACTATTTAAATAACAGATTGGAGACACTTATCTACCGGCA
SPD0150_KO_R2	GGCTTTCGTTTGTAGCGTCA

Supplementary Table 7. X-ray data collection and refinement statistics for PsaA crystals

	Cd^{2+}-PsaA-wild type (4utp)	Cd^{2+}-PsaA-D280N (4uto)
**Data Collection**		
Wavelength (Å)	0.954	0.954
Resolution range (Å)	43.0-2.00 (2.07-2.00)	19.73-1.55 (1.61-1.55)
Space group	$P2_1$	$P1$
**Unit cell parameters**		
a, b, c (Å)	61.2, 48.0, 101.5	39.7, 59.3, 62.5
α, β, γ (°)	90.0, 107.0, 90.0	106.9, 104.9, 93.4
Completeness (%)	93.8 (93.9)	96.6 (94.8)
R_{merge}	0.11 (0.67)	0.13 (0.73)
$< I / \sigma(I)>$	5.24 (3.13)	8.6 (1.9)
Multiplicity	1.9 (1.9)	3.4 (3.4)
**Refinement***		
Resolution range (Å)	43.0-2.00 (2.05-2.00)	19.73-1.55 (1.61-1.55)
No. reflections	34346 (2510)	73161 (3568)
R_{work}/ R_{free} (%)	22.1/26.0	18.1/21.4
**No. of atoms**		
Protein	4518	4448
Ligand/ion	0/25	16/2
Water	124	510
**B-factors (Å^2)**		
Protein	39.3	15.2
Ligand/ion	0/58.9	15.2/14.7
Waters	36.2	28.1
**R.m.s deviations**		
Bond lengths (Å)	0.005	0.011
Bond angles (°)	0.969	1.33

All datasets were collected from single crystals.

* Values in parentheses correspond to the highest resolution shell.
